# The Effectiveness of Dialectical Behavior Therapy in Reducing Self‐Directed Violence and Suicidality in Usual Care Settings: A Meta‐Analysis

**DOI:** 10.1002/jclp.70195

**Published:** 2026-08-01

**Authors:** Colleen A. Maxwell, Katherine E. Holland, Lauren Young, Eunice Y. Chen, Thomas Olino, Philip C. Kendall

**Affiliations:** ^1^ Department of Psychology and Neuroscience Temple University Philadelphia Pennsylvania USA; ^2^ Department of Psychiatry University of Pennsylvania Perelman School of Medicine Philadelphia Pennsylvania USA; ^3^ Department of Child and Adolescent Psychiatry and Behavioral Sciences Children's Hospital of Philadelphia Philadelphia Pennsylvania USA; ^4^ Icahn School of Medicine at Mount Sinai, Center of Excellence in Eating and Weight Disorders New York New York USA; ^5^ Department of Behavioral Health Trinitas Regional Medical Center – Rober Wood Johnson – Barnabus Health Elizabeth New Jersey USA; ^6^ College of Graduate Studies and Research Immaculata University Immaculata Pennsylvania USA

**Keywords:** crisis service use, dialectical behavior therapy, effectiveness research, meta‐analysis, self‐directed violence, suicidality

## Abstract

Dialectical behavior therapy (DBT) has shown efficacy in reducing suicidality, though less is known about its effectiveness when delivered in usual care (UC) settings, where the resource‐intensive nature of the program often results in modifications during implementation. This systematic meta‐analysis examined the effectiveness of DBT in reducing suicidality‐related outcomes when delivered in usual care settings. A systematic search was conducted and studies were identified for inclusion if they evaluated a DBT‐based intervention with original, quantitative data reflecting pre‐ and post‐treatment measurement of at least one of the three suicidality‐related outcomes: (i) self‐directed violence (SDV), (ii) crisis service use (CSU), and (iii) suicidal ideation (SI). Random effects models were used to estimate pooled effects. Meta‐regression analyses were used to compare pooled effects across usual care and non‐usual care settings and to examine the differential effects of theoretical predictors often involved in treatment modification. The final sample included 83 studies. In uncontrolled studies, DBT was associated with a large reduction in SDV and medium reductions in CSU and SI. In controlled studies, DBT demonstrated a significant effect for SDV but not for CSU or SI. Adjustment for publication bias attenuated the CSU findings. No differences were observed between usual care and non‐usual care settings. Substantial heterogeneity was observed across models. In uncontrolled studies, positive associations emerged between therapist training and SDV reduction, adherence reporting and CSU reduction, and outpatient setting and SI reduction. Results provide the strongest support for the effectiveness of DBT in reducing SDV, with more limited and variable evidence for CSU and SI. Results should be interpreted with caution given heterogeneity and evidence of publication bias.

## 1 Introduction

Suicide is a major public health concern, accounting for over 700,000 deaths annually worldwide (World Health Organization 2021). In 2023, the United States' (US) population rate of suicide was 14.1 per 100,000 (Ahmad et al. 2024). National survey data indicate that suicidal thoughts, plans, and attempts have increased in adolescents and adults (McCance‐Katz 2020). Self‐directed violence (SDV), which describes any intentional act of self‐directed harm or injury regardless of suicidal intent, results in a visit to the emergency department at a rate of over 1.6 per 100,000 annually (Canner et al. 2018) and rates of emergency department visits for SDV have further increased since the COVID‐19 pandemic (Yard et al. 2021).

The use of emergency and crisis services represents substantial financial costs to the health care system. In 2013, the direct costs of SDV were estimated at $1.6 billion, with the overall economic costs (including indirect costs such as lost productivity) estimated at $93.5 billion annually in the United States (Shepard et al. 2016). Importantly, individuals who present to the emergency department due to SDV are highly likely to return, with 16%–25% estimated to return within the year (Owens et al. 2002; Saunders and Smith 2016). Moreover, the frequency of SDV episodes is associated with increased service system costs per incident (Sinclair et al. 2011). Correspondingly, the reduction of SDV is a public health priority, with emphasis on efforts targeting individuals engaging in chronic SDV.

Dialectical Behavior Therapy (DBT) is a widely used intervention based in cognitive‐behavioral and eastern philosophical principles that was initially developed for the treatment of chronically suicidal individuals (Linehan 1987; Linehan et al. 1993). At the core of DBT is the concept of the “dialectic” in which individuals are asked to simultaneously hold two competing concepts and to search for the relative truth in each and to find ways in which they can coexist. One key dialectic is the synthesis of acceptance and change. This dialectic involves practicing self‐acceptance and at the same time honoring the need to change behaviors that are life threatening and/or that contribute to suffering. This dialectic is observed in the treatment approach where providers provide high levels of validation and empathy for individuals' experiences of suffering and simultaneously use behavioral interventions to promote new skills that support behavior change.

The foundation of DBT is the biosocial theory, which describes a model for how emotion dysregulation develops and maintains over time (Crowell et al. 2009). In short, biological vulnerability to emotion (which includes emotional sensitivity, emotional reactivity, and slow return to emotional baseline) and trait impulsivity transact with invalidating factors in the environment to shape emotion dysregulation over time. Specifically, this occurs through cyclical patterns in which one learns that escalating emotional responses are more effective in gaining attention or meeting needs than more regulated expressions. Correspondingly, the environment becomes increasingly invalidating to such emotional expressions. Resultantly, individuals are likely to engage in destructive and inadequate coping methods to avoid painful emotional experiences. SDV is thus conceptualized as a form of ineffective coping.

Designed as an outpatient treatment, standard DBT involves four components (group skills training, individual therapy, telephone coaching, and therapist consultation team) that are delivered over 26 weeks (Linehan 2015b, 2015a). Standard DBT also includes a pretreatment orientation period in which providers connect with individuals to give information about treatment, collaboratively identify behavioral targets, and obtain the individual's commitment to treatment. There are four stages in which DBT targets different functional areas: stage one focuses on increasing behavioral control and skills use, stage two addresses trauma and other underlying concerns, stage three focuses on individual goals around self‐respect, and stage four focuses on establishing a greater sense of connectedness and purpose (Swenson et al. 2002). In stage one, life‐threatening behaviors, including SDV of any kind, are explicitly identified as the highest priority treatment target, followed by therapy‐interfering behaviors, quality of life‐interfering behaviors, and skill deficits. Behaviors and skill use are monitored with daily diary cards, which are reviewed weekly with therapists in individual and group sessions. The group component of DBT, commonly considered a critical contributor to treatment outcomes, involves the teaching of four sets of skills: mindfulness, distress tolerance, emotion regulation, and interpersonal effectiveness. Phone coaching enhances skill acquisition by providing access to therapists in moments of crisis in which additional coaching is needed. Individual therapy addresses motivation for change and problem‐solving around specific breakdowns in skills application. Weekly therapist consultation groups provide an opportunity for team members to connect for mutual support given the challenging nature of this work. Because emotion dysregulation and associated SDV behaviors often emerge in adolescence, DBT was adapted for use with adolescents and their families (DBT‐A; Miller et al. 2017; Rathus and Miller 2015).

There is ample evidence that DBT is efficacious in reducing SDV. A meta‐analysis of 18 controlled trials found a pooled effect of 0.32 in reducing SDV and a pooled effect of 0.38 in reducing crisis service use (DeCou et al. 2019). This meta‐analysis included studies with wide variation in terms of sample age, history of SDV, setting, and modified delivery of standard DBT. Another meta‐analysis which examined DBT‐A's efficacy identified seven controlled trials with a pooled effect of 0.44 in reducing SDV compared to control groups and 16 pre‐post evaluations with a pooled effect of 0.98 in reducing SDV (Kothgassner et al. 2021). Meta‐analytic work examining RCTs for SDV more broadly have found similar results (Fox et al. 2020; Kothgassner et al. 2020). Importantly, Fox et al. (2020) found smaller treatment effects across interventions than have been estimated in the past (*g* = 0.11) and limited evidence for DBT's superiority over other treatments. Nevertheless, there was a significant relationship between the use of DBT and treatment effects for self‐harm outcomes and combined outcomes of suicidal thoughts and behaviors.

Despite evidence of its efficacy, DBT is not widely accessible. Most individuals in the United States access mental health care in community mental health clinics (CMHCs), which offer a variety of services (though mostly outpatient) at affordable rates (Garfield 2011). CMHCs, which have been described as “the backbone of the safety net system” (Cummings et al. 2017, p. 481) represent the primary provider for low‐income and underinsured individuals through the use of sliding scale fees and Medicaid, which is the single largest payer of behavioral health services in the US (Smith et al. 2015). Although data suggest that CMHC's are important in suicide prevention (Hung et al. 2020), uptake of DBT in CMHCs has been slow and large‐scale implementation efforts have been stifled by system‐ and organization‐level barriers, particularly related to resource limitations (Carmel et al. 2014; Ditty et al. 2015; Quetsch et al. 2020).

Cost has consistently been identified as a barrier to the large‐scale implementation of empirically supported treatments in CMHCs, where resources are scarce (Beidas et al. 2016; Pegg et al. 2019; Roundfield and Lang 2017). Implementation expenses include direct costs such as training and supervision as well as indirect costs such as time spent on unbillable activities (e.g., therapist consultation team, self‐study, etc.; Lang and Connell 2017). For DBT, financial concerns were identified as a primary barrier to sustainment by team leaders and administrators involved in two large‐scale implementations of DBT in Britain and the United States (Herschell et al. 2009; King et al. 2018). Despite the implementation expenses, it has been posited that DBT may be a cost‐efficient solution where the downstream savings associated with reduction in SDV may justify the up‐front cost of implementation. Indeed, several studies have performed economic evaluations that support this notion (Amner 2012; Brazier et al. 2006; Priebe et al. 2012).

The effectiveness of DBT when delivered in usual care settings is largely unexamined in meta‐analytic work. Though several individual studies have demonstrated that DBT can be effectively implemented in usual care (UC) settings (Berk et al. 2020; Ditty et al. 2015; Pasieczny and Connor 2011; Priebe et al. 2012), most meta‐analyses on DBT have focused on its efficacy. Efficacy studies evaluate treatment effects in highly controlled research settings that can differ from UC contexts in many ways including the training and expertise of the therapists delivering the treatment, treatment fidelity and adherence, and non‐representative study samples (Clarke 1995; Flay 1986). This is contrasted with effectiveness research, which examines outcomes in less controlled conditions, designed to be more representative of UC. To date, no meta‐analysis has specifically examined the effectiveness of DBT when delivered in UC settings. One meta‐analytic review evaluated DBT's effectiveness by examining studies conducted in a variety of settings (e.g. specialty clinics, research clinics, and CMHCs); however, because the sample was not limited to studies in UC and no analyses specifically examined the effects between settings, the conclusions to be drawn about effectiveness in UC settings are limited (DeCou et al. 2019). Further, most meta‐analyses have limited their samples to controlled trials with comparison conditions, excluding evaluations with study designs that are more common or fitting to research in UC (e.g., pre‐post evaluations, open trials). Thus, a meta‐analytic evaluation of DBT's effectiveness in reducing SDV when delivered in UC is a critical next step.

Productive meta‐analytic study of intervention effectiveness requires a method for systematically distinguishing between usual care and research‐based contexts. However, relatively few efforts have attempted to operationalize this distinction in the psychotherapy literature. One of the most influential approaches was proposed by Weisz et al. (1995), who identified a set of practice‐oriented criteria to characterize the extent to which psychotherapy trials reflect real‐world service delivery. These criteria capture whether interventions are tested with clinic‐referred populations, delivered in routine service settings, embedded within ongoing service systems, and implemented by practicing clinicians. Using this framework, prior meta‐analytic work on youth‐oriented psychotherapy found that these dimensions were not consistently associated with differences in treatment outcomes, suggesting that interventions may retain effectiveness under real‐world conditions. Subsequent work has applied similar criteria to specific intervention models. For example, Michelson et al. (2013) examined parent management training across these practice dimensions and likewise found limited and inconsistent evidence that effectiveness varied as a function of real‐world delivery context, although some effects emerged for interventions delivered within established service systems. To date, however, no studies have systematically applied this distinction to dialectical behavior therapy (DBT), despite its extensive evaluation in controlled trials and widespread implementation in routine clinical care. This represents an important gap, as DBT is a complex, resource‐intensive intervention that is often adapted across settings and providers. The practice‐oriented criteria described above are particularly well‐suited to the study of DBT, as they directly index key dimensions of its real‐world implementations including the clinical populations served, the organizational contexts in which care is delivered, and the training and specialization of providers. As such, these criteria offer a useful framework for evaluating whether and how DBT effectiveness generalizes from research settings to routine clinical practice.

Given the resource‐intensive nature of DBT, key elements may be lost in the transition from research to UC contexts, which may influence its effectiveness. One element often adapted is program completeness. For example, clinics may opt to provide a DBT skill training group *or* individual DBT alone, rather than the comprehensive standard DBT program. Though studies have found that the isolated delivery of portions of the program can be effective (Andión et al. 2012; Linehan et al. 2015; Valentine et al. 2015), others have found that outcomes are stronger with the full program (Linehan et al. 2015). Program incompleteness may decrease outcomes because DBT skills are designed to progress along a three‐phase process of acquisition, strengthening, and generalization (Linehan 1993), with various program components designed to support distinct parts of this process (Mattei and Sposato 2020). Skill building may be further affected by reductions in the dose of DBT provided. For example, the prescribed 48‐week dose may be truncated in UC to meet the needs of the community (i.e., by providing four 12‐week groups rather than two 26‐week groups).

Therapist training can impact the translation to UC settings because it has been identified as a central element to DBT outcomes and represents a substantial implementation cost (King et al. 2018; Koerner 2013). The prescribed “gold standard” training regimen is termed the “Intensive Training Model” and includes 10 days of training, divided into two parts with 6 months of self‐study in between (Landes and Linehan 2012). The intensive training model has been evaluated with community‐based therapists and resulted in improvements in self‐reported knowledge, confidence, and use of DBT components; however, only 55% of participants completed training due to high levels of turnover (Herschell et al. 2014). Though this model, which requires substantial investment by CMHCs and therapists, may not fit for UC contexts (Carmel et al. 2014), less intensive 2‐day and online training models have also received some support (Dimeff et al. 2009, 2011; Landes et al. 2016). Similarly, research on DBT in UC has highlighted the utility of ongoing consultation support throughout implementation (Carmel et al. 2014; Herschell et al. 2014; Swain et al. 2010). The present study evaluated (a) the effectiveness of DBT in reducing suicidality‐ and SDV‐related outcomes when delivered in UC settings, (b) whether the extent to which the study is delivered in UC moderates the effect of DBT on these suicidality‐ and SDV‐related outcomes, and (c) whether the theoretical predictors of effectiveness of program completeness, dose, therapist training, and implementation support moderated the effect of DBT on suicidality‐ and SDV‐related outcomes. The relationship of setting and treatment adherence to the effectiveness of DBT in reducing suicidality‐ and SDV‐related outcomes was further explored.

## 2 Methods

### 2.1 Search Strategy and Study Selection

All systematic review and meta‐analytic procedures were preregistered with PROSPERO. A search was conducted for all relevant articles published in English since 1987 (the year of DBT's inception) in PubMed, MEDLINE, and PsyclNFO using the search string: (Dialectical Behav* OR DBT*) AND (Suic* OR NSSI* OR Crisis* OR Emergency*). The electronic database search was supplemented by a manual search of the reference lists of included articles. The initial literature search was conducted in December 2021, with an updated search completed in September 2022. A citation management system (Zotero) eliminated duplicates. All studies were uploaded into Rayyan, a meta‐analytic web‐application that provides a streamlined platform for coding processes (Johnson and Phillips 2018; Ouzzani et al. 2016).

Two researchers independently reviewed all abstracts, meeting iteratively to discuss disagreements. Disagreements were discussed and consensus reached. All reasons for exclusion were documented in accordance with PRISMA guidelines. The studies included after the abstract review were then subjected to a full‐text review and the same procedures for using Rayyan, resolving disagreements, and documenting exclusion were applied. Agreement between coders for the abstract phase was *κ* = 0.88 and for the full‐text coding phase, agreement was *κ* = 0.75.

The inclusion criteria were as follows: (a) the study reported an empirical evaluation of a DBT‐based intervention and (b) the study reported original, quantitative data reflecting pre‐ and post‐treatment measurement of at least one of the three quantitative suicidality‐related outcomes. To meet the first inclusion criterion, the DBT intervention delivered must have taught skills encompassing all four modules of standard DBT (e.g., mindfulness, distress tolerance, emotion regulation, and interpersonal effectiveness) and could be delivered in group and/or individual format. Correspondingly, studies that made adaptations of any kind to standard DBT (e.g., DBT‐A, DBT plus another intervention) were included provided they specifically covered all four skills modules, regardless of format or the use of additional interventions. Study designs included pilot studies, randomized controlled trials (RCTs), quasi‐experimental studies, wait‐list controls, and open trials. To meet the second inclusion criterion of reporting quantitative data reflecting pre‐ and post‐treatment measurement of suicidality‐related outcomes, studies must have included a measure of at least one of the following: (a) self‐directed violence (SDV) which may include suicidal and/or non‐suicidal self‐injurious behavior (NSSI), (b) use of psychiatric crisis services (e.g., emergency department visits, inpatient hospitalizations), or (c) suicidal ideation (SI).

Accordingly, the following studies were excluded: meta‐analyses, reviews, protocol papers, single subject designs/case studies, nonempirical work such as commentaries, measure development studies, studies reporting only qualitative outcomes, studies not intervening on the individual of interest (e.g., parent interventions without any youth involvement for the treatment of youth suicidality). Studies in which the sample included incarcerated participants were also excluded. Unpublished dissertations were included due to the evidence suggesting that the methodological quality is comparable to that of peer‐reviewed, published studies and to avoid publication bias (McLeod and Weisz 2004). Studies that reported secondary analyses on data previously reported in a separate main outcomes paper were excluded so as not to artificially inflate the meta‐analytic results with duplicate data. Similarly, dissertations that reported data later published in a peer reviewed journal were excluded and the peer reviewed study reporting that data was included. Studies that reported only long‐term follow‐up outcome data were also excluded. There were no exclusion criteria based on age or diagnosis.

### 2.2 Data Extraction and Coding

The included studies (*n* = 83) were divided between the two researchers for data extraction. A subset of 20% of the final set was coded by both researchers and evaluated for agreement to verify consistency in the data‐extraction process. Data extraction was conducted using REDCap data management system.

#### 2.2.1 Intervention

DBT intervention was coded as a dichotomous variable (0/1) for the presence of each of the components of standard DBT (e.g., group skills training, individual therapy, phone coaching, and consultation team). The dose of individual and group portions was quantified as the prescribed dose per study procedures and calculated as a continuous variable in the form of hours. When the amount of time was not specified, the prescribed time according to the manual was used (e.g., 2.5‐h weekly skills group, 1‐h weekly individual sessions). The presence of intervention components outside of the defined parameters for DBT was also coded dichotomously (0/1), including additional DBT‐related intervention provided as part of study procedures (e.g., mindfulness groups), additional non‐DBT services provided as part of study procedures (e.g., psychiatric consultation, medication management, prolonged exposure). Of note, although stage two of DBT involves addressing underlying problems such as trauma, the current study included primarily stage one DBT interventions due to the nature of the specified outcomes. As such, inclusion of specific interventions for secondary concerns (such as trauma) were coded as “non‐DBT” services. Studies were also coded dichotomously (0/1) for the assessment of adherence and dropout. Data for both variables were extracted from studies that included it. Agreement between coders for dose was ICC = 0.94 (*r* = 0.96) and for program completeness ICC = 0.91 (*r* = 0.92).

#### 2.2.2 Setting

The setting in which DBT was delivered was coded as (a) residential, (b) inpatient, (c) partial hospitalization, (d) intensive outpatient, (e) outpatient, (f) emergency room/crisis setting (preadmission), or (g) other. The country in which each study took place was also noted.

#### 2.2.3 Therapists

Descriptive information about the service providers was recorded including data on previous experience and training background. Descriptions of the specific training in the delivery of DBT provided to therapists was also recorded; a continuous variable of the number of hours spent in training was calculated for studies that reported it. A dichotomous variable was coded (0/1) to indicate if study therapists or organizations received any additional implementation support (e.g., ongoing consultation with a training body). Agreement between coders for therapist training was ICC = 0.99 (*r* = 1.00) and for implementation support, *κ* = 0.91.

#### 2.2.4 Usual Care (Uc)

In line with previous meta‐analyses (Michelson et al. 2013; Weisz et al. 1995), four key criteria that define UC were used. Each was coded dichotomously (0/1). Studies failing to report necessary information to discern a criterion were coded 0, to be conservative. A total score was summed from each of these four criteria to create a UC score (0–4). Agreement between coders for the total UC score was ICC = 0.84 (*r* = 0.84).

The first criterion is that the sample was clinic‐referred. Clinic‐referred samples describe participants who were seeking services and recruited through typical referral streams (e.g., by their primary care physician). Public advertisements or other methods of community outreach do not fulfill this criterion. Second, the intervention was delivered in a routine setting. A routine setting is a place in which care is typically delivered (e.g., hospital, community mental health clinic, emergency room, etc.). Settings in which the provision of services was specifically created for the purpose of the research study (e.g., university research clinic) do not fulfill this criterion. Third, the intervention was delivered as a part of routine service. A routine service describes a pre‐established service‐provision framework independent of the research study. Hybrid clinical research contexts, such as research hospitals, can fulfill this criterion if services are routinely provided outside of the context of clinical research (e.g., an individual is not required to participate in research to receive care). Fourth, the providers were not research therapists, rather, they were professional, practicing clinicians pre‐embedded in the service delivery setting independent of the research study. Non‐research therapists routinely deliver care in the routine service setting as a part of their role in the organization. Thus, providers that do not fulfill this criterion include treatment developers or researchers working directly under them, study authors or staff, or therapists provided by the research study. A university faculty member may be considered a nonresearch therapist if their role in the study is based on their clinical position in the clinical service setting and they are not directly involved in the research study in any way. Trainees may fulfill this criterion if their role in the service setting is due to routine clinical training.

#### 2.2.5 Participants

Descriptions of the demographics of study participants were recorded for each study including gender, age, race/ethnicity, socioeconomic status, and important clinical characteristics such as psychopathology. Inclusion criteria were also recorded.

#### 2.2.6 Outcomes

Three domains of suicidality‐related outcomes were coded for each study. The first was self‐directed violence (SDV) which included suicidal and/or non‐suicidal self‐injurious behavior (NSSI). Though there are valuable distinctions between suicidal behavior and NSSI in terms of intention and lethality, these two categories of behavior were grouped together because they share common risk factors (e.g., borderline personality disorder, impulsivity), both tend to increase with acquired capability (i.e., habituation to fear and pain associated with self‐directed violence), and both often occur within the context of intrapersonal distress (Hamza et al. 2012). Correspondingly, when present, SDV behavior is the primary behavioral target of DBT. The second outcome domain is psychiatric crisis service use (CSU), such as emergency department (ED) visits and inpatient hospitalizations. The third outcome domain is suicidal ideation (SI), a strong predictor of suicidal behavior (Victor and Klonsky 2014).

### 2.3 Statistical Analyses

#### 2.3.1 Effect Size Estimation

In accordance with recommendations by Borenstein et al. (2009), effect sizes for controlled and uncontrolled studies were calculated and analyzed separately on the basis that their results reflect fundamentally different questions and account for different sources of bias. For controlled studies, Cohen's d_s_ effect size (standardized mean difference) was calculated as the difference between the post‐treatment mean scores of the DBT and non‐DBT comparison groups divided by the pooled standard deviation (Cohen 1992). In the two instances in which studies included more than one non‐DBT comparison group (Gregory and Sachdeva 2016; Yang et al. 2020), the comparison group that most closely resembled other comparison groups was chosen. For example, Gregory and Sachdeva (2016) compared a DBT to dynamic deconstructive psychotherapy and treatment as usual (TAU); in this case, the TAU comparison group was selected as the comparator for effect size calculations given that the modal type of comparison group across controlled studies was an active usual care comparator. For uncontrolled studies using within‐subjects or pre/post designs, Cohen's d_av_ effect size (standardized mean gain) was calculated as the difference between the mean pre‐ and post‐treatment scores divided by the average standard deviation of both measures (Cumming 2012; Lakens 2013). Several studies compared the outcomes of multiple DBT conditions but did not include a non‐DBT comparison group. When conditions were defined based on a manipulation of the intervention delivery format, setting, or dose, each condition was treated as an individual uncontrolled pre/post study. When conditions were defined based on participant characteristics rather than a manipulation of the intervention, the two conditions were combined and treated as an uncontrolled pre/post study.

To adjust for measurement error, an artifact distribution correction (Schmidt and Hunter 2015) was applied using available alpha statistics for the measurement tools used through the “psychmeta” package in R Studio statistical software (Dahlke and Wiernik 2019). For studies that did not report reliability, the reliability provided in the measurement tool development paper was used. Mean imputation was used for studies that did not report any reliability and for which the tool used did not have a psychometric paper available to reference. To account for small samples, all Cohen's *d* effect sizes were converted to Hedges' *g*, which involves the computation of a correction factor between 0 and 1 that is based on sample size, so that larger samples have a *g* value similar to the original *d* value (Hedges and Olkin 1985). Outcomes reported as binary proportions or odds ratios were transformed directly to Hedges' *g* (Lipsey and Wilson 2001).

For studies that reported multiple indices of a single outcome domain, the effect sizes were aggregated assuming a correlation of rho = 0.6, so as not to overrepresent results from studies that included a greater number of outcome indices. The aggregated effect sizes for each domain were then weighted by multiplying the inverse of the squared standard error (SE), yielding a singular final weighted effect size estimate for each outcome domain reported by each study.

To evaluate the pooled effect of DBT on each outcome when delivered in the context of UC, the subset of studies with a UC score of 4 were selected. In anticipation of a high degree of between‐study heterogeneity, a random effects model (METHOD = “REML”) using the “metafor” package in R studio statistical software (Viechtbauer 2010) was used to pool effect sizes. This process was repeated for each outcome domain using separate models for controlled and uncontrolled studies (6 models total). Knapp‐Hartung adjustments (Knapp and Hartung 2003) for random effects models were used to calculate confidence intervals around the pooled effect. For models estimating the pooled effect of uncontrolled studies, three‐level hierarchical models were fitted to account for nesting due to the fact that studies comparing multiple DBT conditions were treated individually (Cheung 2013). The fit of the three‐level models was compared with the fit of the two‐level models that did not account for nesting to examine the utility of this additional parameter. The heterogeneity between effect sizes was examined using Cochran's Q, a statistic used for determining if the studies sampled were drawn from different populations (Cochran 1954). Heterogeneity between studies was further quantified using the *I* ^2^ index, which describes the proportion of variance that is attributable to true differences in effect size in relation to overall variance (Higgins et al. 2003; Higgins and Thompson 2002). *I* ^2^ values are interpreted as low, moderate, and substantial at 25%, 50%, and 75%, respectively.

#### 2.3.2 Publication Bias

Several steps were taken to minimize risk of publication bias, or the error that is introduced by the phenomenon that studies with significant results may be more likely to be published than studies without significant results. First, unpublished dissertations were included and examined for differences in effect size in comparison to published studies. Second, in line with the Cochrane risk‐of‐bias methodology and previous meta‐analyses (Weisz et al. 2013), studies were assessed on three criteria: (1) random sequence generation, (2) participant blindness to condition, and (3) attrition rate. The attrition rate variable was dichotomized into studies with greater than (0) and less than 40% attrition (1). Participant blindness to condition was not present in any of the studies examined and thus dropped as an indicator of publication bias. Effect sizes were examined to determine if they differed in relation to the two remaining criteria (Weisz et al. 2013). Additionally, Eggers's regressions (Sterne and Egger 2005) were conducted to estimate publication bias and using the trim‐and‐fill method (Duval and Tweedie 2000), adjusted effect sizes were computed to correct for funnel plot asymmetry.

#### 2.3.3 Subgroup Analysis and Meta‐Regression

To evaluate whether UC criteria moderate the effect of DBT on suicidality‐ and SDV‐related outcomes, the full sample of studies was dichotomized into two groups representing UC scores = 4 and UC score < 4. Using a fixed effects plural model in accordance with statistical procedures by Borenstein and Higgins (2013) via the “metafor” package in R studio statistical software (Viechtbauer 2010), a pooled effect was calculated for each group and the effects of the subgroups were compared.

Given the high degree of variability in DBT's implementation in UC settings, analyses were conducted to determine whether the theoretical predictors of effectiveness of program completeness, dose, therapist training, and implementation support moderated the effect of DBT on suicidality‐ and SDV‐related outcomes. Intervention setting and treatment adherence were additionally included as exploratory variables. To evaluate these relationships, all categorical predictors were dummy coded and meta‐regression models using Restricted Maximum Likelihood estimation were run for each of the three outcomes in two steps (Viechtbauer 2005). First, each predictor was entered into the model individually, then all predictors were entered into a multiple meta‐regression model simultaneously. In the case that exceedingly high levels of multicollinearity were detected among predictors (*r* ≥ 0.8) the models were adjusted by eliminating redundant predictors.

## 3 Results

### 3.1 Study Characteristics

The study selection process and reasons for exclusion appear in the PRISMA diagram (Figure 1). A total of 872 nonduplicate articles were identified through the database and manual searches. Six‐hundred and fifty‐one records were excluded in the abstract review yielding 221 articles for full‐text review. One hundred and thirty‐eight of those were excluded leaving a total of 83 studies for the meta‐analysis. A table summarizing the 83 studies included in the final sample can be found in Table 1.

**Figure 1:**
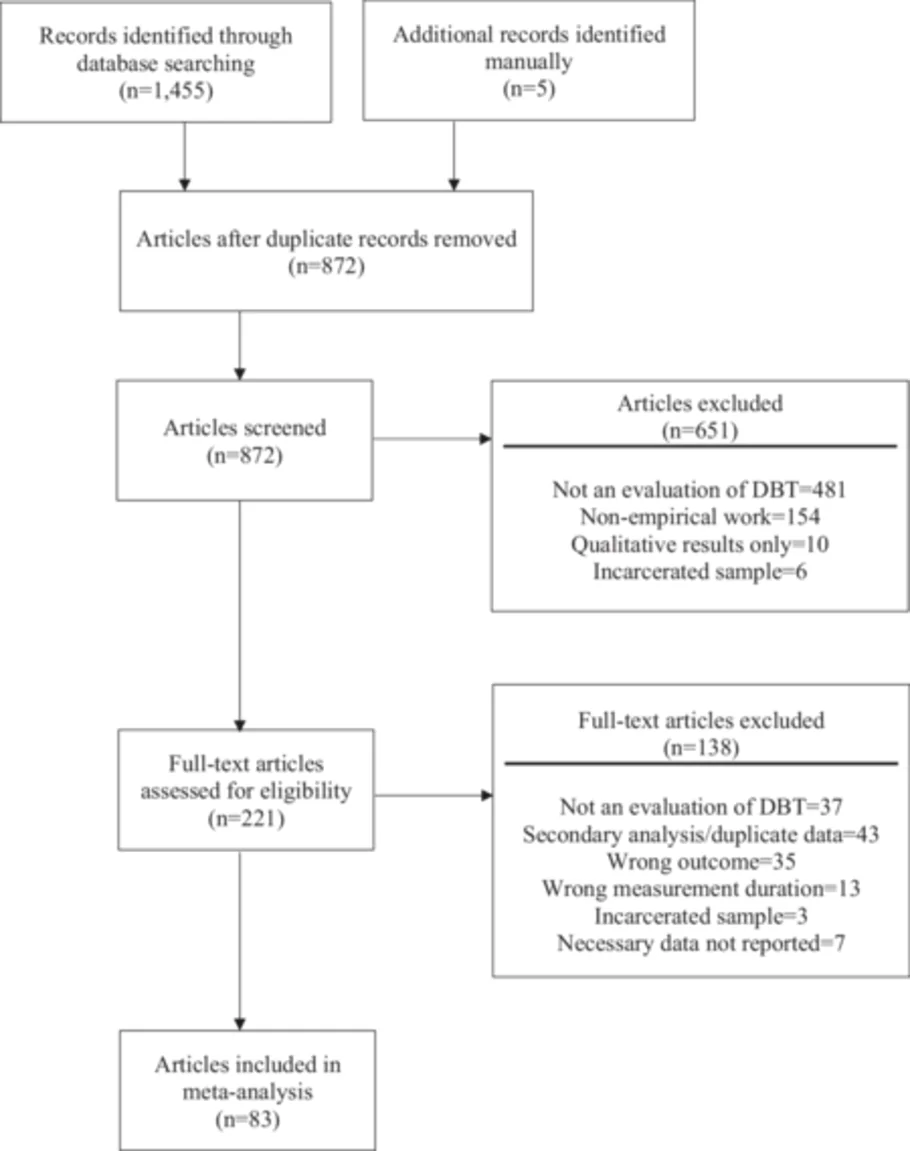
PRISMA diagram displaying study selection process and reasons for article exclusion.

**Table 1:** Summary of studies analyzed (*n* = 83).

References	Study design	Setting	Usual care criteria	Sample	DBT intervention	Outcomes collected	Therapist training
Andión et al. (2012)	CCT^a^ comparing two DBT conditions: (a) Combined individual DBT and DBT skills group (b) Individual DBT only	Spain Single‐site Outpatient	Total Score = 3 Criteria Met: C1 – Clinic Referred Sample C2 – Routine Setting C3 – Routine Service	*N* = 51 100% female Age: 25.6 (6.5) No race/ethnicity data reported	(a) Combined individual + skills group Individual: 48 h over 48 weeks Group: 96 h over 48 weeks Phone coaching provided Consultation team held (b) Individual DBT only Individual: 48 h over 48 weeks Phone coaching provided	SDV: 2 Proportion with full reduction of suicide attempts (present at pre and no longer present at post) Proportion with full reduction of NSSI (present at pre and no longer present at post) CSU: 1 Proportion with full reduction of visits to ED (present at pre and no longer present at post)	Format: Official 10‐day intensive DBT training Hours: 80
Andreasson et al. (2016)	RCT Active alternative comparison: Collaborative Assessment and Management of Suicidality	Denmark Single‐site Outpatient	Total Score = 4 Criteria Met: C1 – Clinic Referred Sample C2 – Routine Setting C3 – Routine Service C4 – Nonresearch Therapists	*N* = 108 74.1% female Age: 31.7 (12.7) No race/ethnicity data reported	Individual: 16 h over 16 weeks Group: 32 h over 16 weeks Phone coaching provided Consultation team held	SDV: 3 Suicide attempt frequency NSSI frequency Self‐harm composite frequency SI: 1 Beck Scale for Suicide Ideation	Format: Official 10‐day intensive DBT training Hours: 80
Apsche et al. (2006)	RCT Active alterative comparison: Mode Deactivation therapy	US Single‐site Residential	Total Score = 4 Criteria Met: C1 – Clinic Referred Sample C2 – Routine Setting C3 – Routine Service C4 – Nonresearch Therapists	*N* = 20 0% female Age: 16 (NR), range 15‐18 Race/ethnicity: 55.0% African American 35.0% White 10.0% Hispanic /Latino American	Did not report specific details of any components of DBT delivery; provided services for up to 1 year with no specific dose prescribed	SI: 1 Suicidal Ideation Questionnaire‐High School (SIQ‐HS)	Format: “at the official DBT training center” Hours: NR
Barnicot and Crawford (2019)	CCT Active alterative comparison: Mentalisation‐based therapy	UK Multi‐site Outpatient	Total Score = 4 Criteria Met: C1 – Clinic Referred Sample C2 – Routine Setting C3 – Routine Service C4 – Nonresearch Therapists	*N* = 90 72.0% female Age: 31.0 (13.0) Race/ethnicity: 64.0% White 36.0% Black and minority ethnicity	Individual: 52 h over 52 weeks Group: NR hours over 52 weeks Phone coaching provided Consultation team held	SDV: 1 Self‐harm frequency CSU: 2 A&E (ED) frequency Psychiatric hospital admission frequency	Format: NR Hours: NR
Berk et al. (2020)	Uncontrolled, pre‐post design	US Single‐site Outpatient	Total Score = 4 Criteria Met: C1 – Clinic Referred Sample C2 – Routine Setting C3 – Routine Service C4 – Nonresearch Therapists	*N* = 24 92.0% female Age: 15.2 (1.3) Race/ethnicity: 63.0% Latino 17.0% Caucasian 21.0% Other	Individual: NR hours over 26 weeks Group: 32.5 h over 26 weeks Phone coaching provided Consultation team held Additional DBT provided: Family and collateral sessions provided as needed	SDV: 2 Suicide attempt frequency NSSI frequency CSU: 2 ED visit frequency Hospitalization frequency SI: 1 Suicide Ideation Questionnaire Junior (SIQ‐JR)	Format: 2‐day training by first author Hours: 16
Blennerhassett et al. (2009)	Uncontrolled, pre‐post design	Ireland Multi‐site Outpatient	Total Score = 3 Criteria Met: C1 – Clinic Referred Sample C2 – Routine Setting C3 – Routine Service	*N* = 10 100% female Age: 29.4 (NR), range 18‐44 No race/ethnicity data reported	Individual: NR hours over 26 weeks Group: 52 h over 26 weeks Phone coaching provided Consultation team held	SDV: 1 Frequency of self‐harm episodes	Format: Majority of therapists received intensive 10‐day training Hours: 80
Bohus et al. (2000)	Uncontrolled, pre‐post design	US Single‐site Inpatient	Total Score = 1 Criteria Met: C1 – Clinic Referred Sample	*N* = 24 100% female Age: 28.3 (7.0) No race/ethnicity data reported	Individual: NR hours over 12 weeks Group: 18 h over 12 weeks Consultation team held Additional DBT provided: Milieu skills coaching, weekly homework group, weekly fundamentals group	SDV: 1 Proportion of group with NSSI	Format: NR Hours: NR
Bohus et al. (2004)	CCT Passive treatment as usual comparison condition	Germany Single‐site Inpatient	Total Score = 2 Criteria Met: C1 – Clinic Referred Sample C4 – Nonresearch Therapists	*N* = 60 100% female Age: 29.5 (6.51) No race/ethnicity data reported	Individual: 24 h over 12 weeks Group: 24 h over 12 weeks Phone coaching provided Consultation team held Additional DBT provided: Once weekly mindfulness group	SDV: 1 Frequency of self‐mutilating behavior	Format: NR Hours: NR
Brüne et al. (2013)	Uncontrolled, pre‐post design	Germany Single‐site Inpatient	Total Score = 4 Criteria Met: C1 – Clinic Referred Sample C2 – Routine Setting C3 – Routine Service C4 – Nonresearch Therapists	*N* =16 100% female Age: 27.2 (9.5) No race/ethnicity data reported	Individual: NR hours over 6 weeks Group: 8 h over 4 weeks Phone coaching provided Consultation team held Additional DBT provided: Mindful attention groups held twice weekly	SDV: 1 Borderline Symptom List (BSL)–Severity of Self‐Injurious Behavior subscale	Format: NR Hours: NR
Buerger et al. (2019)	Uncontrolled, pre‐post design	Germany Multisite Outpatient	Total Score = 4 Criteria Met: C1 – Clinic Referred Sample C2 – Routine Setting C3 – Routine Service C4 – Nonresearch Therapists	*N* = 72 91.7% female Age: 15.7 (1.2) No race/ethnicity data reported	Individual: 20.75 h over 25 weeks (50‐min sessions) Group: 40 h over 20 weeks Phone coaching provided Additional DBT provided: Family therapy every FOUR sessions of individual therapy	SDV: 2 Self‐harm frequency Suicide attempt frequency	Format: NR Hours: NR
Chang et al. (2021)	Uncontrolled, pre‐post design	Taiwan Single‐site Outpatient	Total Score = 4 Criteria Met: C1 – Clinic Referred Sample C2 – Routine Setting C3 – Routine Service C4 – Nonresearch Therapists	*N* = 18 77.8% female Age: 31.3 (7.3) No race/ethnicity data reported	Individual: 52 h over 52 weeks Group: 130 h over 52 weeks Phone coaching provided Consultation team held	SDV: 1 Frequency of parasuicides score using BSL‐Supplement for Assessing Behavior SI: 1 Beck Scale for Suicide Ideation	Format: Official 10‐day intensive DBT training plus additional training Hours: 128
Chen et al. (2008)	Uncontrolled, pre‐post design	US Single site Outpatient	Total Score = 0	*N* = 8 100% female Age: 31 (NR), range 24‐56 Race/ethnicity: 87.5% Caucasian 12.5% Korean American	Individual: NR hours over 24 weeks Group: NR hours over 24 weeks Phone coaching provided Consultation team held Additional DBT provided:	SDV: 1 Individual frequency of SDV (NSSI + suicide attempts)	Format: NR Hours: NR
Comtois et al. (2007)	Uncontrolled, pre‐post design	US Single‐site Outpatient	Total Score = 4 Criteria Met: C1 – Clinic Referred Sample C2 – Routine Setting C3 – Routine Service C4 – Nonresearch Therapists	*N* = 23 96.0% female Age: 34 (NR), range 19‐54 No race/ethnicity data reported	Individual: 52 h over 52 weeks Group: 156 h over 52 weeks Phone coaching provided Consultation team held	SDV: 1 Medically‐treated parasuicide frequency CSU: 5 Psychiatric‐related ED visit frequency Psychiatric inpatient hospital admissions frequency Crisis‐related inpatient admission frequency Number of psychiatric‐related inpatient days Number of crisis systems engaged	Format: Official 10‐day intensive DBT training Hours: 80
Courtney and Flament (2015)	Uncontrolled, pre‐post design	Canada Single‐site Outpatient	Total Score = 4 Criteria Met: C1 – Clinic Referred Sample C2 – Routine Setting C3 – Routine Service C4 – Nonresearch Therapists	*N* = 41 93.4% female Age: 16.5 (0.8) No race/ethnicity data reported	Individual: 14 h over 14 weeks Group: 14 weeks; hours per week not reported Phone coaching provided Consultation team held Additional DBT provided: One parent‐only group session	SDV: 1 Proportion of group with an incident of self‐harm SI: 1 Suicidal Ideation Questionnaire (SIQ)	Format: Formally trained in DBT with at least 6 days' worth of training Hours: 48
Cowperthwait (2017)	CCT^a^ comparing two DBT conditions: (a) Multifamily skills group (b) Parallel family skills group	US Single site Outpatient	Total Score = 1 Criteria Met: C1 – Clinic Referred Sample	*N* = 37 94.6% female 3.4% male Age: 15.8 (1.1) Race/ethnicity: 83.8% White 13.5% Black 2.7% Multiracial	Both conditions received the same amount of DBT intervention: Individual: 21.58 h over 26 weeks Group: 39 h over 26 weeks Phone coaching provided Consultation team held	SDV: 3 NSSI Frequency NSSI Severity SA frequency CSU: 1 Frequency of hospitalizations	Format: Official 10‐day intensive DBT training Hours: 80
Decker et al. (2019)	Uncontrolled, pre‐post design	US Single‐site Outpatient	Total Score = 2 Criteria Met: C2 – Routine Setting C3 – Routine Service	*N* = 17 35% female 65% male Age: NR Race/ethnicity: 76% Caucasian 18% African American 1% Other	Group: 52 h over 26 weeks	SI: 2 Suicidal Behaviors Questionnaire Beck Scale for Suicidal Ideation	Format: NR Hours: NR
Druhn (2010)	Uncontrolled, pre‐post design	US Single‐site IOP	Total Score = 4 Criteria Met: C1 – Clinic Referred Sample C2 – Routine Setting C3 – Routine Service C4 – Nonresearch Therapists	*N* = 36 100% female Age: 38.4 (8.6) Race/ethnicity: 89.0% White 2.8% American Indian 2.8% Asian 5.5% Unknown	Individual: NR hours over 48 weeks Group: 396 h over 48 weeks (3x 2.75‐h skills group sessions)	SDV: 2 Self‐injurious behavior frequency Suicide attempt frequency CSU: 1 Psychiatric hospitalization frequency	Format: NR Hours: NR
Edwards et al. (2021)	Uncontrolled, pre‐post design	US Single‐site IOP	Total Score = 4 Criteria Met: C1 – Clinic Referred Sample C2 – Routine Setting C3 – Routine Service C4 – Nonresearch Therapists	*N* = 56 78.6% female Age not reported Race/ethnicity: 89.3% White/Caucasian 5.4% Hispanic 1.8% Black/African American 3.6% Asian	Group: 64 h over 16 weeks (2x 2‐h skills group sessions) Phone coaching provided	SDV: 2 Suicidal behavior frequency Self‐harm frequency	Format: NR Hours: NR
Federici (2010)	Uncontrolled, pre‐post design	Canada Single‐Site Outpatient	Total Score = 3 Criteria Met: C2 – Routine Setting C3 – Routine Service C4 – Nonresearch Therapists	*N* = 31 97% female 3% male Age: 33.6 (11.8) No race/ethnicity data reported	Group: 40 h over 20 weeks Consultation team held	SDV: 2 Frequency, duration, and severity of self‐harm measured by the Deliberate Self‐Harm Inventory Proportion of group with an incident of self‐harm	Format: NR Hours: NR
Feigenbaum et al. (2012)	RCT Active treatment as usual comparison group	UK Single‐site Outpatient	Total Score = 4 Criteria Met: C1 – Clinic Referred Sample C2 – Routine Setting C3 – Routine Service C4 – Nonresearch Therapists	*N* = 41 73.2% female Age: 35.1 (7.6) No race/ethnicity data reported	Individual: 48 h over 48 weeks Group: 120 h over 48 weeks Phone coaching provided Consultation team held	SDV: 2 Deliberate self‐harm frequency Suicide attempt frequency CSU: 2 Inpatient psychiatric hospital days A&E (ED) visit frequency SI: 1 Overt Aggression Scale–Suicidality subscale	Format: 2‐day workshop plus official 10‐day intensive DBT training Hours: 96
Fischer and Peterson (2015)	Uncontrolled, pre‐post design	US Single site Outpatient	Total Score = 2 Criteria Met: C2 – Routine Setting C3 – Routine Service	*N* = 10 100% female Age: 16.2 (1.0) Race/ethnicity: 100% White	Individual: 26 h over 26 weeks Group: 52 h over 26 weeks Phone coaching provided Consultation team held	SDV: 1 Frequency of NSSI	Format: NR Hours: NR
Fleischhaker et al. (2011)	Uncontrolled, pre‐post design	Germany Single site Outpatient	Total Score = 3 Criteria Met: C1 – Clinic Referred Sample C2 – Routine Setting C3 – Routine Service	*N* = 12 100% female Age: NR No race/ethnicity data reported	Individual: 24 h over 24 weeks Group: 48 h over 24 weeks Phone coaching provided	SDV: 1 Proportion of sample with NSSI	Format: NR Hours: NR
Flynn et al. (2017)	Uncontrolled, pre‐post design	Ireland Multisite (national effort) Outpatient	Total Score = 4 Criteria Met: C1 – Clinic Referred Sample C2 – Routine Setting C3 – Routine Service C4 – Nonresearch Therapists	*N* = 71 86.0% female Age: 40.0 (9.8) No race/ethnicity data reported	Individual: 52 h over 52 weeks Group: 130 h over 52 weeks Phone coaching provided Consultation team held	SI: 1 Beck Scale for Suicide Ideation	Format: Official 10‐day intensive DBT training Hours: 80
Flynn et al. (2019)	Uncontrolled, pre‐post design	Ireland Multisite (national effort) Outpatient	Total Score = 4 Criteria Met: C1 – Clinic Referred Sample C2 – Routine Setting C3 – Routine Service C4 – Nonresearch Therapists	*N* = 70 85.0% female Age: 15.7(1.1) No race/ethnicity data reported	Individual: 16 h over 16 weeks Group: 32 h over 16 weeks Phone coaching provided Consultation team held	SDV: 2 Proportion of group with an incident of self‐harm Self‐harm frequency CSU: 3 ED visit frequency Acute inpatient admission frequency Number of inpatient days SI: 1 Questionnaire for Suicidal Ideation (self‐developed)	Format: Official 10‐day intensive DBT training plus additional teaching Hours: 160
Flynn et al. (2020)	Uncontrolled, pre‐post design	Ireland Multisite (national effort) Outpatient	Total Score = 4 Criteria Met: C1 – Clinic Referred Sample C2 – Routine Setting C3 – Routine Service C4 – Nonresearch Therapists	*N* = 196 80.6% female Age: range 18‐64 with 60% between 25 and 44 No race/ethnicity data reported	Individual: 48 h over 48 weeks Group: 48 h over 48 weeks Phone coaching provided Consultation team held	SDV: 2 Proportion of group with an incident of self‐harm Self‐harm frequency CSU: 2 Inpatient hospital admission frequency ED visit frequency SI: 1 Questionnaire for Suicidal Ideation (self‐developed)	Format: Official 10‐day intensive DBT training Hours: 70
Geddes et al. (2013)	Uncontrolled, pre‐post design	Australia Single‐site Outpatient	Total Score = 4 Criteria Met: C1 – Clinic Referred Sample C2 – Routine Setting C3 – Routine Service C4 – Nonresearch Therapists	*N* = 6 100% female Age: 15.1 (NR) No race/ethnicity data reporte	Individual: 26 h over 26 weeks Group: 36 h over 18 weeks Phone coaching provided Consultation team held	SDV: 1 Proportion of group with an incident of self‐harm SI: 1 Proportion of group reporting the presence of SI	Format: One day of “in house” DBT training Hours: 8
Gillespie et al. (2019)	CCT^a^ comparing two DBT conditions: (a) 16 weeks of treatment (b) 24 weeks of treatment	Ireland Multisite (national effort) Outpatient	Total Score = 4 Criteria Met: C1 – Clinic Referred Sample C2 – Routine Setting C3 – Routine Service C4 – Nonresearch Therapists	*N* = 48 85.0% female Age: 15.6 (1.2) No race/ethnicity data reported	(a) 16 weeks of treatment Individual: 16 h over 16 weeks Group: 32 h over 16 weeks Phone coaching provided Consultation team held (b) 24 weeks of treatment Individual: 24 h over 24 weeks Group: 48 h over 24 weeks Phone coaching provided Consultation team held	SDV: 2 Proportion of group with an incident of self‐harm Self‐harm frequency SI: 1 Questionnaire for Suicidal Ideation (self‐developed)	Format: NR Hours: NR
Goldstein et al. (2007)	Uncontrolled, pre‐post design	US Single‐site Outpatient	Total Score = 3 Criteria Met: C1 – Clinic Referred Sample C2 – Routine Setting C3 – Routine Service	*N* = 10 80% female 20% male Age: 15.8 (1.5) Race/ethnicity: 60.0% White 10.0% Black 3.0% Multiracial	Individual: 18 h over 48 weeks Group: 36 h over 48 weeks Phone coaching provided	SDV: 1 Any NSSI ‐ measured by KSADS DRS Item 30 SI: 2 Modified Scale for Suicidal Ideation (MSSI) KSADS‐DRS item 25	Format: Two 2‐day DBT training workshops conducted by a certified Behavior Tech trainer Hours: 32
Goldstein et al. (2015)	RCT Active treatment as usual comparison group	US Single‐site Outpatient	Total Score = 4 Criteria Met: C1 – Clinic Referred Sample C2 – Routine Setting C3 – Routine Service C4 – Nonresearch Therapists	*N* = 20 75.0% female Age: 16.1 (1.9) Race/ethnicity: 80.0% Caucasian 10.0% African American 20.0% Multiracial	Individual: 18 h over 48 weeks Group: 18 h over 48 weeks Phone coaching provided Consultation team held	SDV: 2 Proportion of group with any NSSI incident Proportion of group with any suicide attempt incident SI: 1 Suicidal Ideation Questionnaire – Junior (SIQ‐JR)	Format: Didactic Hours: 16
Goodman et al. (2016)	RCT Passive treatment as usual comparison group	US Single‐site Outpatient	Total Score = 4 Criteria Met: C1 – Clinic Referred Sample C2 – Routine Setting C3 – Routine Service C4 – Nonresearch Therapists	*N* = 91 33.3% female Age: 38.3 (10.9) Race/ethnicity: 14.3% White (non‐Hispanic) 31.9% Black (non‐Hispanic) 1.1% American Indian 1.1% Asian/Pacific Islander 45.1% Hispanic 6.6% Multiracial	Individual: 26 h over 26 weeks Group: 39 h over 26 weeks Phone coaching provided Consultation team held	SDV: 1 Proportion of group with any suicide attempt incident SI: 1 Beck Scale for Suicide Ideation	Format: Official 10‐day intensive DBT training Hours: 80
Gregory and Sachdeva (2016)	CCT comparing DBT to two non‐DBT comparison groups (a) Active alternative comparison: Dynamic deconstructive psychotherapy (b) Active treatment as usual comparison group^c^	US Multisite Outpatient	Total Score = 3 Criteria Met: C1 – Clinic Referred Sample C2 – Routine Setting C3 – Routine Service	*N* = 41 79.4% female 19.1% male Age: 31.5 (11.7) Race/ethnicity: 88.2% White	Individual: NR hours over 48 weeks Group: NR hours over 48 weeks Phone coaching provided Consultation team held	SDV: 2 NSSI frequency SA frequency	Format: Official 10‐day intensive DBT training Hours: 80
Guillén Botella et al. (2021)	CCT Active alterative comparison: Systems Training for Emotional Predictability and Problem‐solving	Spain Multisite Outpatient	Total Score = 4 Criteria Met: C1 – Clinic Referred Sample C2 – Routine Setting C3 – Routine Service C4 – Nonresearch Therapists	*N* = 72 94.8% female Age: 32.1 (8.83) No race/ethnicity data reporte	Individual: 24 h over 24 weeks Group: 48 h over 24 weeks Phone coaching provided Consultation team held	SI: 1 Suicide Risk Scale (SRS)	Format: NR Hours: NR
Gutteling et al. (2012)	Uncontrolled, pre‐post design	Netherlands Single‐site Outpatient	Total Score = 4 Criteria Met: C1 – Clinic Referred Sample C2 – Routine Setting C3 – Routine Service C4 – Nonresearch Therapists	*N* = 34 100% female Age: 27.3 (NR), range 19‐40 No race/ethnicity data reported	Individual: NR hours over NR weeks Group: 216 h over 48 weeks Phone coaching provided Consultation team held Additional DBT provided: DBT Group Therapy (DBT‐GT)	SI: 3 BDI: Item 9, suicidal thoughts SCL‐90: Item 15, suicidal thoughts SCL‐90: Item 59, thoughts about death	Format: Official 10‐day intensive DBT training Hours: 80
Harley et al. (2007)	Uncontrolled, pre‐post design	US Single‐site Outpatient	Total Score = 4 Criteria Met: C1 – Clinic Referred Sample C2 – Routine Setting C3 – Routine Service C4 – Nonresearch Therapists	*N* = 49 91.8% female Age: 39.0 (10.3) Race/ethnicity: 96.0% White	Group: 52.5 h over 30 weeks Consultation team held	SDV: 1 Personality Assessment Inventory, Borderline Scale, Self‐Harm Subscale SI: 1 Personality Assessment Inventory–Suicide scale	Format: NR Hours: NR
Harned et al. (2012)	Uncontrolled, pre‐post design	US Single‐site Outpatient	Total Score = 4 Criteria Met: C1 – Clinic Referred Sample C2 – Routine Setting C3 – Routine Service C4 – Nonresearch Therapists	*N* = 13 100% female Age: 39.4 (11.4) Race/ethnicity: 69.2% White	Individual: 48 h over 48 weeks Group: 120 h over 48 weeks Phone coaching provided Consultation team held	SDV: 1 Intentional self‐injury acts SI: 1 Suicidal ideation	Format: Official 10‐day intensive DBT training Hours: 80
Harned et al. (2014)	CCT^a^ comparing two DBT conditions: (a) Standard DBT (b) Standard DBT + Prolonged Exposure	US Single‐site Outpatient	Total Score = 4 Criteria Met: C1 – Clinic Referred Sample C2 – Routine Setting C3 – Routine Service C4 – Nonresearch Therapists	*N* = 26 100% female Age: 32.6 (12.0) Race/ethnicity: 80.8% Caucasian 15.4% Biracial 3.8% Asian American	Both conditions received the same amount of DBT intervention: Individual: 48 h over 48 weeks Group: 120 h over 48 weeks Phone coaching provided Consultation team held	SDV: 2 Frequency of NSSI Frequency of SA	Format: Official 10‐day intensive DBT training Hours: 80
Heerebrand et al. (2021)	Uncontrolled, pre‐post design	Australia Single‐site Outpatient	Total Score = 4 Criteria Met: C1 – Clinic Referred Sample C2 – Routine Setting C3 – Routine Service C4 – Nonresearch Therapists	*N* = 114 92.1% female Age: 32.00 (NR), range 19‐63 Race/ethnicity: 86.0% Non‐indigenous Australian 0.8% Indigenous Australian 2.6% European 4.4% Asian 3.5% Another ethnicity 2.6% Unknown	Group: 50 h over 20 weeks Consultation team held	CSU: 2 ED visit frequency Number of inpatient hospital bed days	Format: Official 10‐day intensive DBT training Hours: 80
Hjalmarsson et al. (2008)	Uncontrolled, pre‐post design	Sweden Multisite Outpatient	Total Score = 4 Criteria Met: C1 – Clinic Referred Sample C2 – Routine Setting C3 – Routine Service C4 – Nonresearch Therapists	*N* = 27 100% female Age: 20.2 (5.6) No race/ethnicity data reported	Individual: 48 h over 48 weeks Group: 144 h over 48 weeks Phone coaching provided Consultation team held	SDV: 1 Frequency of parasuicides	Format: 110 h of theory in DBT, 153 h of supervision and 51 h of seminars and workshops Hours: 314
A. C. James et al. (2008)	Uncontrolled, pre‐post design	UK Multisite Outpatient	Total Score = 4 Criteria Met: C1 – Clinic Referred Sample C2 – Routine Setting C3 – Routine Service C4 – Nonresearch Therapists	*N* = 16 100% female Age: NR (NR), range 15‐18 No race/ethnicity data reported	Individual: 48 h over 48 weeks Group: 72 h over 48 weeks Phone coaching provided	SDV: 1 Frequency of episodes of deliberate self‐harm	Format: NR Hours: NR
James et al. (2011)	Uncontrolled, pre‐post design	UK Multisite Outpatient	Total Score = 4 Criteria Met: C1 – Clinic Referred Sample C2 – Routine Setting C3 – Routine Service C4 – Nonresearch Therapists	*N* = 25 88% female 8.3% Male Age: 15.5 (1.5) No race/ethnicity data reported	Individual: 48 h over 48 weeks Group: 96 h over 48 weeks Phone coaching provided Consultation team held	SDV: 1 Frequency of episodes of self‐harm	Format: NR Hours: NR
S. James et al. (2015)	Uncontrolled, pre‐post design	US Single‐site IOP	Total Score = 4 Criteria Met: C1 – Clinic Referred Sample C2 – Routine Setting C3 – Routine Service C4 – Nonresearch Therapists	*N* = 99 84.8% female Age: 14.9 (1.3) Race/ethnicity: 59.0% White 14.1% African American 21.2% Hispanic 6.1% Asian or other	Individual: 12 h over 16 weeks Group: 96 h over 16 weeks Additional DBT provided: 1‐h weekly parent education groups	SDV: 1 Single item assessing frequency of self‐injury using Youth Outcomes Questionnaire‐Self Report (YOQ‐SR)	Format: Official 10‐day intensive DBT training Hours: 80
Katz et al. (2004)	CCT Active treatment as usual comparison	Canada Single‐site Inpatient	Total Score = 4 Criteria Met: C1 – Clinic Referred Sample C2 – Routine Setting C3 – Routine Service C4 – Nonresearch Therapists	*N* = 62 83.9% female Age: 15.4 (NR), range 14‐17 Race/Ethnicity: 72.6% White 1.6% Latino 4.8% Asian/Pacific Islander 19.4% First Nations Populations 1.6% Other background	Individual: 4 sessions of NR hours over 2 weeks Group: 10 sessions of NR hours over 2 weeks Consultation team held Additional DBT provided: Milieu DBT on residential floor	SDV: 1 Parasuicide frequency CSU: 2 Hospitalization frequency ED visit frequency SI: 1 Suicidal Ideation Questionnaire (SIQ)	Format: NR Hours: NR
Koons et al. (2001)	CCT Active treatment as usual comparison	US Single‐site Outpatient	Total Score = 3 Criteria Met: C1 – Clinic Referred Sample C2 – Routine Setting C3 – Routine Service	*N* = 20 100% female Age: 35.0 (7.02) Race/ethnicity: 75% Caucasian 25% African American	Individual: 26 h over 26 weeks Group: 39 h over 26 weeks Phone coaching provided Consultation team held	SDV: 1 Frequency of parasuicides CSU: 1 Psychiatric inpatient admissions SI: 1 Beck Scale for Suicide Ideation	Format: Official 10‐day intensive DBT training Hours: 80
Lakeman et al. (2020)	Uncontrolled, pre‐post design	Australia Single‐site Outpatient	Total Score = 4 Criteria Met: C1 – Clinic Referred Sample C2 – Routine Setting C3 – Routine Service C4 – Nonresearch Therapists	*N* = 22 81.8% female Age: 20.0 (2.5) No race/ethnicity data reported	Individual: 20 h over 20 weeks Group: 60 h over 20 weeks Phone coaching provided Consultation team held Additional DBT provided: Invited to continue in second cycle of skills group	CSU: 2 ED visit frequency Number of hospital admission days	Format: A 5‐day intensive training event was facilitated by an experienced accredited DBT therapist Hours: 40
Leerer (1997)	Uncontrolled, pre‐post design	US Single‐site Inpatient	Total Score = 3 Criteria Met: C1 – Clinic Referred Sample C2 – Routine Setting C3 – Routine Service	*N* = 14 100% female Age: 31.9 (6.7) Race/ethnicity: 100% Caucasian	Group: 55 h over 55 weeks	SDV: 1 Frequency and severity of parasuicidal acts	Format: Official 10‐day intensive DBT training Hours: 80
Lin et al. (2019)	CCT Active alternative comparison: Cognitive therapy group	Taiwan Multisite Outpatient	Total Score = 0	*N* = 82 87.8% female 12.2% male Age: 20.4 (0.7) No race/ethnicity data reported	Group: 16 h over 8 weeks	SDV: 1 Frequency of suicide attempts SI: 1 Adult Suicidal Ideation Questionnaire ‐ Short (ASIQ‐S)	Format: NR Hours: NR
Linehan (1991)	CCT Passive treatment as usual comparison	US Single‐site Outpatient	Total Score = 3 Criteria Met: C1 – Clinic Referred Sample C2 – Routine Setting C3 – Routine Service	*N* = 44 100% female Age: NR No race/ethnicity data reported	Individual: 48 h over 48 weeks Group: 120 h over 48 weeks Phone coaching provided Consultation team held	SDV: 1 Frequency of parasuicidal acts	Format: NR Hours: NR
Linehan et al. (2006)	CCT Active treatment as usual comparison	US Single‐site Outpatient	Total Score = 4 Criteria Met: C1 – Clinic Referred Sample C2 – Routine Setting C3 – Routine Service C4 – Nonresearch Therapists	*N* = 101 100% female Age: 29.3 (7.5) Race/ethnicity: 87.0% White 5.0% Other 4.0% Black 2.0% Asian 1.0% Native American or Alaskan Native	Individual: 48 h over 48 weeks Group: 120 h over 48 weeks Phone coaching provided Consultation team held	SDV: 2 Frequency of suicide attempts Frequency of NSSI SI: 1 Suicidal Behaviors Questionnaire	Format: DBT seminar followed by supervised practice Hours: 45
Linehan et al. (2015)	CCT^a^ comparing three DBT conditions: (a) Standard DBT (b) Individual DBT only (c) DBT skills group only	US Single‐site Outpatient	Total Score = 0	*N* = 99 100% female Age: 30.3 (8.9) Race/ethnicity: 71.0% White 22.0% Biracial 5.0% Asian American 2.0% Other	(a) Standard DBT Individual: 48 h over 48 weeks Group: 120 h over 48 weeks Phone coaching provided Consultation team held (b) Individual DBT only Individual: 48 h over 48 weeks Phone coaching provided Consultation team held (c) DBT skills group only Group: 120 h over 48 weeks Phone coaching provided Consultation team held	SDV: 2 Frequency of SA Frequency of NSSI CSU: 4 Frequency of ED visits for psychiatric reasons Frequency of ED visits for suicidality Frequency of admissions for psychiatric reasons Frequency of admissions for suicidality SI: 1 Suicidal Behaviors Questionnaire	Format: NR Hours: NR
Lynch et al. (2020)	RCT Passive treatment as usual comparison	UK Multisite Outpatient	Total Score = 3 Criteria Met: C1 – Clinic Referred Sample C2 – Routine Setting C3 – Routine Service	*N* = 250 66% female Age: NR Race/ethnicity: 97.0% Caucasian	Individual: 29 h over 29 weeks Group: 67.5 h over 27 weeks Consultation team held Additional DBT provided: Radical openness modules	SI: 1 Modified Scale for Suicide Ideation	Format: Official 10‐day intensive DBT training Hours: 80
Lyng et al. (2019)	CCT^a^ 2 DBT comparison groups: (a) Standard DBT (b) DBT Skills group only	Ireland Multisite Outpatient	Total Score = 4 Criteria Met: C1 – Clinic Referred Sample C2 – Routine Setting C3 – Routine Service C4 – Nonresearch Therapists	*N* = 88 83.0% female Age: 33.3 (9.1) No race/ethnicity data reported	(a) Standard DBT Individual: 24 h over 24 weeks Group: 60 h over 24 weeks Phone coaching provided Consultation team held (b) DBT skills group only Group: 65 h over 26 weeks Consultation team held	SI: 1 Beck Scale for Suicide Ideation	Format: Official 10‐day intensive DBT training Hours: 80
Manning (1997)	CCT^b^ comparing two DBT conditions based on participant characteristics: (a) BPD (b) BPD and comorbid major affective disorder	US Single‐site Outpatient	Total Score = 4 Criteria Met: C1 – Clinic Referred Sample C2 – Routine Setting C3 – Routine Service C4 – Nonresearch Therapists	*N* = 34 100% female Age: 38.8 (NR), range 24‐53 No race/ethnicity data reported	Individual: NR hours over 48 weeks Group: NR weeks 48 h per week not reported Consultation team held	SI: 1 Beck Scale for Suicide Ideation	Format: Investigator‐led training, who had completed DBT intensive training Hours: NR
McCauley et al. (2018)	RCT Active alternative comparison: Individual and Group Supportive Therapy (IGST)	US Multisite Outpatient	Total Score = 1 Criteria Met: C1 – Clinic Referred Sample	*N* = 173 94.8% female Age: 14.89 (1.47) Race/ethnicity: 56.4% White 27.5% Hispanic 7.0% African American 5.9% Asian American 2.3% Other 0.6% Native American	Individual: NR hours over 26 weeks Group: NR hours over 26 weeks Phone coaching provided Consultation team held Additional DBT provided: Parents were seen individually in session 1 and offered 7 or more family sessions	SDV: 2 Proportion of sample with SA Proportion of sample with NSSI SI: 1 SIQ‐Jr	Format: Attended a multiday training led by the treatment developer Hours: NR
McDonell et al. (2010)	CCT Active treatment as usual comparison	US Single‐site Long‐term inpatient	Total Score = 4 Criteria Met: C1 – Clinic Referred Sample C2 – Routine Setting C3 – Routine Service C4 – Nonresearch Therapists	*N* = 155 58.0% female Age: 15.54 (1.20) No race/ethnicity data reported	Consultation team held Additional DBT provided: Milieu DBT provided on inpatient floor	SDV: 1 NSSI frequency	Format: NR Hours: NR
McMain et al. (2009)	RCT Active alternative comparison: General psychiatric management	Canada Multisite Outpatient	Total Score = 4 Criteria Met: C1 – Clinic Referred Sample C2 – Routine Setting C3 – Routine Service C4 – Nonresearch Therapists	*N* = 180 86.1% female Age: 30.4 (9.9) No race/ethnicity data reported	Individual: 48 h over 48 weeks Group: 96 h over 48 weeks Phone coaching provided Consultation team held	SDV: 2 Suicidal and self‐injurious episode frequency Maximum medical risk score for suicide and self‐injurious episodes using Suicide Attempt Self‐Injury interview CSU: 3 ED visit frequency ED visits for suicidal behavior frequency Number of psychiatric hospital bed days	Format: Official 10‐day intensive DBT training Hours: 80
McMain et al. (2017)	RCT Passive treatment as usual comparison	Canada Single‐site Outpatient	Total Score = 4 Criteria Met: C1 – Clinic Referred Sample C2 – Routine Setting C3 – Routine Service C4 – Nonresearch Therapists	*N* = 84 78.6% female Age: 29.7 (8.6) No race/ethnicity data reported	Group: 40 h over 20 weeks Consultation team held	SDV: 2 Suicide or self‐harm frequency (clinician report) Suicide or self‐harm frequency (self‐report) CSU: 2 Proportion of group with any ED visit Proportion of group with any hospitalization incident	Format: NR Hours: NR
Mehlum et al. (2014)	RCT Active treatment as usual comparison	Norway Multisite Outpatient	Total Score = 4 Criteria Met: C1 – Clinic Referred Sample C2 – Routine Setting C3 – Routine Service C4 – Nonresearch Therapists	*N* = 77 88.3% female Age: 15.6 (1.5) Race/ethnicity: 84.9% Norwegian	Individual: 19 h over 19 weeks Group: 38 h over 19 weeks Phone coaching provided Consultation team held Additional DBT provided: Family therapy as needed	SDV: 1 Self‐harm frequency SI: 1 Suicidal Ideation Questionnaire‐Junior (SIQ‐JR)	Format: Intensive training with an additional 12 months of supervised practice on clinical training cases Hours: 80
Meyers et al. (2014)	Uncontrolled, pre‐post design	US Single‐site Outpatient	Total Score = 4 Criteria Met: C1 – Clinic Referred Sample C2 – Routine Setting C3 – Routine Service C4 – Nonresearch Therapists	*N* = 41 46.3% female Age: 47.1 (10.7) Race/ethnicity: 78.0% Caucasian 7.0% African–American 2.0% Asian 12.0% Unknown/Refused	Individual: 26 h over 26 weeks Group: 52 h over 26 weeks Phone coaching provided Consultation team held	CSU: 2 ED visit frequency Partial day hospitalization frequency	Format: Official 10‐day intensive DBT training or a day‐long overview of DBT philosophy and skills followed by a 6‐month 90‐min weekly didactic seminar Hours: 63.5
Meyers et al. (2017)	Uncontrolled, pre‐post design	US Single‐site IOP	Total Score = 4 Criteria Met: C1 – Clinic Referred Sample C2 – Routine Setting C3 – Routine Service C4 – Nonresearch Therapists	*N* = 33 48.5% female Age: 43.21 (9.92) Race/ethnicity: 75.8% Caucasian 15.2% Native American 3.0% Asian American 3.0% African American 3.0% Other race	Individual: NR hours over 12 weeks Group: NR hours over 12 weeks Additional DBT provided: Weekly mindfulness practice groups Weekly interpersonal skills practice groups	SI: 1 Beck Scale for Suicide Ideation	Format: NR Hours: NR
Navarro‐Haro et al. (2018)	CCT Active alternative comparison: Cognitive‐behavioral program for Eating Disorders	Spain Multisite Other setting: Combination of outpatient and day hospital (PHP)	Total Score = 3 Criteria Met: C1 – Clinic Referred Sample C2 – Routine Setting C3 – Routine Service	*N* = 118 100% female Age: 27.4 (8.75) No race/ethnicity data reported	Individual: 26 h over 26 weeks Group: 48 h over 24 weeks Phone coaching provided Consultation team held	SDV: 2 Frequency of suicide attempts Frequency of NSSI episodes CSU: 1 Frequency of hospitalizations	Format: NR Hours: 40
Pasieczny and Connor (2011)	CCT Passive treatment as usual comparison	Australia Single‐site Outpatient	Total Score = 4 Criteria Met: C1 – Clinic Referred Sample C2 – Routine Setting C3 – Routine Service C4 – Nonresearch Therapists	*N* = 90 93.3% female Age: 33.6 (10.1) No race/ethnicity data reported	Individual: 26 h over 26 weeks Group: 52 h over 26 weeks Phone coaching provided Consultation team held	SDV: 2 Suicide attempt frequency Self‐harm frequency CSU: 3 ED visit frequency Psychiatric admission frequency Number of psychiatric hospital bed days SI: 1 Beck Scale for Suicide Ideation	Format: Basic training from experienced local trainers Hours: 32
Pistorello et al. (2012)	RCT Active treatment as usual comparison	US Single‐site Outpatient	Total Score = 3 Criteria Met: C1 – Clinic Referred Sample C2 – Routine Setting C3 – Routine Service	*N* = 63 81% female 19% male Age: 20.9 (1.9) Race/ethnicity: 69.8% White 11.1% Hispanic 6.3% Asian/Asian American 4.8% Native American 3.2% African American 4.8% Other	Individual: 36.52 h over 44 weeks Group: 36 h over 24 weeks Phone coaching provided Consultation team held Additional DBT provided: As‐needed family interventions	SDV: 3 Proportion of group with any occurrence of NSSI (interview) Proportion of group with any occurrence of NSSI (self‐report) Proportion of group with any occurrence of suicide attempt (interview) SI: 1 Suicidal Behaviors Questionnaire (SBQ‐23)	Format: NR Hours: 30
Prendergast and McCausland (2007)	Uncontrolled, pre‐post design	Australia Single‐site Outpatient	Total Score = 4 Criteria Met: C1 – Clinic Referred Sample C2 – Routine Setting C3 – Routine Service C4 – Nonresearch Therapists	*N* = 16 100.0% female Age: 36.4 (7.4) No race/ethnicity data reported	Individual: 30 h over 24 weeks (60‐90‐min sessions, averaged to 75 min) Group: 60 h over 24 weeks Phone coaching provided Consultation team held	SDV: 3 Parasuicidal/suicidal frequency Medical severity of average parasuicidal/suicidal incident Suicidal intent of average parasuicidal/suicidal incident CSU: 2 Psychiatric hospital admission frequency Number of psychiatric hospital bed days	Format: Official 10‐day intensive DBT training Hours: 80
Priebe et al. (2012)	RCT Passive treatment as usual comparison	UK Single‐site Outpatient	Total Score = 4 Criteria Met: C1 – Clinic Referred Sample C2 – Routine Setting C3 – Routine Service C4 – Nonresearch Therapists	*N* = 80 87.5% female Age: 32.2 (10.8) Race/ethnicity: 57.5% White 15.0% Black 21.3% Asian 6.3% Mixed/Other	Individual: 48 h over 48 weeks Group: 96 h over 48 weeks Phone coaching provided	SDV: 1 Self‐harm frequency	Format: All therapists were trained by the treatment developer's official training provider Hours: NR
Ramsey et al. (2021)	RCT^b^ comparing two DBT conditions: (a) Standard DBT‐A (b) DBT‐A plus 10‐15‐min cognitive intervention	US Single‐site Partial hospitalization	Total Score = 3 Criteria Met: C2 – Routine Setting C3 – Routine Service C4 – Nonresearch Therapists	*N* = 40 75% female 25% male Age: 14.9 (1.9) Race/ethnicity: 92.5% White 5.0% African American 2.5% Asian American 2.5% Hispanic	Both conditions received the same amount of DBT intervention: Individual: 7 h over 7 weeks Group: 21 h over 7 weeks Phone coaching provided Consultation team held	SDV: 1 Frequency of NSSI	Format: Foundational Training in DBT Hours: 40
Rathus and Miller (2002)	CCT Active treatment as usual comparison	US Single‐site Outpatient	Total Score = 3 Criteria Met: C1 – Clinic Referred Sample C2 – Routine Setting C3 – Routine Servic	*N* = 111 78.4% female Age: 15.3 (1.7) Race/ethnicity: 67.6% Hispanic 17.1% African American 8.1% White 0.9% Asian American 6.3% Other	Individual: NR hours over 12 weeks Group: NR hours over 12 weeks Consultation team held	SDV: 1 Proportion of group with a suicide attempt CSU: 1 Proportion of group with psychiatric hospitalization	Format: NR Hours: 16
Rizvi et al. (2016)	Uncontrolled, pre‐post design	US Single‐site Outpatient	Total Score = 3 Criteria Met: C1 – Clinic Referred Sample C2 – Routine Setting C3 – Routine Service	*N* = 16 75% female Age: 27.5 (7.7) Race/ethnicity: 68.8% White 12.5% Non‐White Hispanic 12.5% Multiracial 6.3% Black	Individual: NR hours over 26 weeks Group: NR hours over 26 weeks Phone coaching provided Consultation team held	SI: 1 Urge to self‐harm	Format: NR Hours: NR
Rizvi et al. (2017)	Uncontrolled, pre‐post design	US Single‐site Outpatient	Total Score = 4 Criteria Met: C1 – Clinic Referred Sample C2 – Routine Setting C3 – Routine Service C4 – Nonresearch Therapists	*N* = 50 80.0% female Age: 29.5 (9.6) Race/ethnicity: 68.0% Caucasian 16.0% Mixed race 6.0% non‐White Hispanic 4.0% Asian 4.0% Black 2.0% Other	Individual: 26 h over 26 weeks Group: 52 h over 26 weeks Phone coaching provided Consultation team held	SDV: 2 Suicide attempt frequency NSSI frequency	Format: Official 10‐day intensive DBT training Hours: 80
Robinson et al. (2018)	Uncontrolled, pre‐post design	Canada Single‐site Outpatient	Total Score = Criteria Met: C1 – Clinic Referred Sample C2 – Routine Setting C3 – Routine Service C4 – Nonresearch Therapists	*N* = 97 89.7% female No age or race/ethnicity data reported	Individual: NR hours over 48 weeks Group: 96 h over 48 weeks Phone coaching provided Consultation team held	CSU: 3 Proportion of group with any crisis service use Proportion of group with any ED visit Proportion of group with any inpatient hospitalization	Format: Official 10‐day intensive DBT training Hours: 80
Santamarina‐Perez et al. (2020)	Uncontrolled, pre‐post design	Spain Single‐site Outpatient	Total Score = 3 Criteria Met: C1 – Clinic Referred Sample C2 – Routine Setting C3 – Routine Service	*N* = 35 88.6% female Age: 15.3 (1.33) No race/ethnicity data reported	Individual: 12 h over 16 weeks Group: 16 h over 16 weeks Phone coaching provided Additional DBT provided: Parents attended separate skills group	SDV: 1 NSSI frequency SI: 1 SIQ‐JR	Format: NR Hours: 40
Sinnaeve et al. (2018)	RCT^a^ comparing two DBT conditions: (a) Standard DBT (b) Step‐down DBT	Netherlands Multisite Other: (a) Outpatient (b) Combination of 3 months inpatient and 9 months outpatient	Total Score = 4 Criteria Met: C1 – Clinic Referred Sample C2 – Routine Setting C3 – Routine Service C4 – Nonresearch Therapists	*N* = 84 95.0% female Age: 25.9 (6.8) No race/ethnicity data reported	(a) Standard DBT Individual: NR hours over 48 weeks Group: NR hours over 48 weeks Phone coaching provided Consultation team held (b) Step‐down DBT Individual: NR hours over 48 weeks Group: NR hours over 48 weeks Phone coaching provided Consultation team held Additional DBT provided: Daily mindfulness classes Weekly group sessions on validation skills and chain analyses	SDV: 3 Self‐injury with suicidal intent frequency Self‐injury with ambivalent intent frequency NSSI frequency	Format: NR Hours: 24
Sparapani (2015)	Uncontrolled, pre‐post design	US Single‐site Outpatient	Total Score = 4 Criteria Met: C1 – Clinic Referred Sample C2 – Routine Setting C3 – Routine Service C4 – Nonresearch Therapists	*N* = 34 No gender, age, or race/ethnicity data reported	Individual: Variable, no specific dose prescribed Group: Variable, no specific dose prescribed Phone coaching provided Consultation team held	SI: 1 Self‐developed SI questionnaire	Format: Official 10‐day intensive DBT training Hours: 80
Stanley et al. (2007)	Uncontrolled, pre‐post design	US Multisite Outpatient	Total Score = 0	*N* = 20 85% female Age: 32.2 (8.7) Race/ethnicity: 85% Caucasian	Individual: NR hours over 26 weeks Group: NR hours over 26 weeks Phone coaching provided Consultation team held	SDV: 2 NSSI episode frequency NSSI urge frequency SI: 1 Suicidal ideation reported via diary card	Format: NR Hours: NR
Stepp et al. (2008)	Uncontrolled, pre‐post design	US Single‐site Outpatient	Total Score = 4 Criteria Met: C1 – Clinic Referred Sample C2 – Routine Setting C3 – Routine Service C4 – Nonresearch Therapists	*N* = 27 85% female Age: 30.40 (NR), range 16‐61 Race/ethnicity: 96.0% Caucasian	Individual: NR hours over 48 weeks Group: NR hours over 48 weeks Phone coaching provided Consultation team held	SDV: 1 Personality Assessment Inventory–Borderline Scale, Self‐Harm Subscale (PAI BOR‐S)	Format: NR Hours: NR
Stiglmayr et al. (2014)	Uncontrolled, pre‐post design	Germany Multisite Outpatient	Total Score = 3 Criteria Met: C2 – Routine Setting C3 – Routine Service C4 – Nonresearch Therapists	*N* = 78 91.5% female 8.5% male Age: 30.1 (8.1) No race/ethnicity data reported	Individual: 39.84 h over 48 weeks Group: 96 h over 48 weeks Phone coaching provided Consultation team held Additional DBT provided: 5 initial sessions prior to 12‐month program	SDV: 1 NSSI frequency CSU: 2 Frequency of inpatient stays	Format: Training provided by certified DBT training institute Hours: 64
Turner (2000)	RCT Active treatment as usual comparison	US Single‐site Outpatient	Total Score = 4 Criteria Met: C1 – Clinic Referred Sample C2 – Routine Setting C3 – Routine Service C4 – Nonresearch Therapists	*N* = 24 79.2% female Age: 22.0 (NR), range 18‐27 Race/ethnicity: 79.2% Caucasian 16.7% African American 4.2% Asian American	Individual: NR hours over 49 weeks Consultation team provided	SDV: 2 Severity of parasuicidal behavior rating (0‐8) Suicidal/self‐harm frequency CSU: 1 Frequency of hospitalization bed days SI: 1 Beck Scale for Suicide Ideation	Format: 12 90‐min training sessions over 3 months Hours: 18
Van Dijk et al. (2013)	Uncontrolled, pre‐post design	Canada Single‐site Outpatient	Total Score = 3 Criteria Met: C1 – Clinic Referred Sample C2 – Routine Setting C3 – Routine Service	*N* = 75 75% female 25% male Age: 43.3 (11.1) No race/ethnicity data reported	Group: 18 h over 12 weeks	CSU: 2 Frequency of ED visits Frequency of hospitalization	Format: NR Hours: NR
Verheul et al. (2003)	RCT Passive treatment as usual comparison	Netherlands Single‐site Outpatient	Total Score = 1 Criteria Met: C1 – Clinic Referred Sample	*N* = 64 100% female Age: 34.9 (7.7) Race/ethnicity: 97% Dutch nationality	Individual: NR hours over 48 weeks Group: 108 h over 48 weeks Phone coaching provided Consultation team held	SDV: 2 Proportion of group with any suicide attempt Proportion of group with any NSSI	Format: NR Hours: NR
Walton et al. (2020)	RCT Active treatment as usual comparison	Australia Single‐site Outpatient	Total Score = 4 Criteria Met: C1 – Clinic Referred Sample C2 – Routine Setting C3 – Routine Service C4 – Nonresearch Therapists	*N* = 162 77.0% female Age: 26.6 (7.8) Race/ethnicity: 86.0% White Caucasian 6.0% Aboriginal 8.0% Other	Individual: 48 h over 48 weeks Group: 120 h over 48 weeks Phone coaching provided Consultation team held	SDV: 1 Suicidal/nonsuicidal self‐injury frequency	Format: Official 10‐day intensive DBT training Hours: 80
Wetterborg et al. (2020)	Uncontrolled, pre‐post design	Sweden Multisite Outpatient	Total Score = 4 Criteria Met: C1 – Clinic Referred Sample C2 – Routine Setting C3 – Routine Service C4 – Nonresearch Therapists	*N* = 30 0.0% female Age: 35.3 (8.6) No race/ethnicity data reported	Individual: 48 h over 48 weeks Group: 120 h over 48 weeks	SDV: 2 Proportion of group with any suicide attempt Self‐harm frequency	Format: NR Hours: NR
Williams et al. (2010)	CCT^a^ comparing two DBT conditions: (a) DBT skills group + DBT individual therapy (b) DBT skills group + non‐DBT individual therapy	Australia Single‐site Outpatient	Total Score = 4 Criteria Met: C1 – Clinic Referred Sample C2 – Routine Setting C3 – Routine Service C4 – Nonresearch Therapists	*N* = 140 85.7% female Age: 35.9 (10.6) No race/ethnicity data reported	(a) DBT skills group + DBT individual therapy Individual: NR hours over 20 weeks Group: 40 h over 20 weeks Consultation team held (b) DBT skills group + non‐DBT individual therapy Group: 40 h over 20 weeks Consultation team held	CSU: 3 Number of inpatient psychiatric hospital bed days ED visit frequency Number of calls to crisis service line	Format: NR Hours: NR
Woodberry and Popenoe (2008)	Uncontrolled, pre‐post design	US Single‐site Outpatient	Total Score = 3 Criteria Met: C1 – Clinic Referred Sample C2 – Routine Setting C3 – Routine Service	*N* = 46 89.0% female Age: 16.0 (1.4) Race/ethnicity: 96.0% Caucasian	Individual: NR hours over 15 weeks Group: 26.25 h over 15 weeks Phone coaching provided Consultation team held Additional DBT provided: Family therapy as needed	SI: 2 Frequency rating for “want to hurt self” item on Trauma Symptom Checklist for Children (TSCC) Frequency rating for “want to kill self” item on Trauma Symptom Checklist for Children (TSCC)	Format: Introductory and advanced trainings through Behavior Tech Hours: 16
Yang et al. (2020)	RCT comparing DBT to two non‐DBT comparison groups (a) Active treatment as usual comparison group^c^ (b) Wait list	US Single‐site Outpatient	Total Score = 0	*N* = 65 43.3% female Age: 19.20 (0.8) No race/ethnicity data reported	Group: 24 h over 12 weeks Phone coaching provided	SI: 1 Suicidal Behaviors Questionnaire‐Revised (SBR‐Q)	Format: NR Hours: NR

Abbreviations: BPD = borderline personality disorder, CCT = controlled clinical trial, CSU = crisis service use, DBT = dialectical behavior therapy, ED = emergency department, IOP = intensive outpatient program, NR = not reported, NSSI = nonsuicidal self‐injury, PHP = partial hospitalization program; RCT=randomized controlled trial, SA = suicide attempt, SDV = self‐directed violence, SI = suicidal ideation.

^a^ Indicates that DBT conditions were treated as individual effects in uncontrolled designs.

^b^ Indicates comparison groups were combined.

^c^ Indicates comparison group that was used for analyses.

With respect to their design, 41 studies (49.4%) were uncontrolled pre/post studies that did not include a comparison group. Thirty studies (36.1%) used controlled designs in which the outcomes of DBT condition were compared to at least one non‐DBT comparison condition. Of the 30 controlled studies, the most common form of non‐DBT comparison condition was an active UC comparator (*n* = 14) in which the study procedures specifically provided services that were designed to be representative of the typical services that would be provided in the clinical setting in which the study took place. Eight studies used passive UC comparators in which the services were not specifically provided as part of the study, but referrals to UC service streams were provided and/or engagement in other services was encouraged. Eight studies used comparison conditions that represented an alternative specialty treatment protocol. Of the 30 controlled studies, just over half (*n* = 16) were RCTs and the other half were unrandomized clinical controlled trials (*n* = 14).

Twelve studies (14.5%) compared two or more DBT conditions. James et al. (2015) and Manning (1997) defined conditions based on the participant characteristics (insurance status and diagnosis, respectively) rather than a manipulation of the intervention; thus, the outcomes were averaged and treated as a single condition, uncontrolled design. Ten (12.0%) of the studies compared two or more DBT conditions by manipulating the intervention delivery in some way. Of note, the study by Ramsey et al. (2021) did not report adequate information to analyze the conditions separately; thus, they were combined and treated as a single uncontrolled design condition. Because each DBT condition in nine of the ten studies was treated as an individual, uncontrolled design nested within study cluster, there were a total of 92 total effect sizes (k; also referred to as cases) analyzed in the full sample with 62 treated as data from uncontrolled designs and 30 treated as data from controlled designs.

Regarding individual UC criteria, 73 studies (88.0%; *k* = 80, 87.0%) met criterion one of a clinic‐referred sample, criterion two (*k* = 79, 86.0%) of taking place in a routine setting, and criterion three (*k* = 79, 86.0%) of being delivered as part of a routine service. Notably, all studies that met criterion two also met criterion three. Fewer studies (*n* = 56, 67.5%; *k* = 61, 66.3%) met criterion four of being delivered by non‐research therapists. Regarding UC scores, 52 studies (62.7%; *k* = 57, 62.0%) met all four UC criteria and categorized in the “High UC” subgroup. Thirty‐one studies (37.3%; *k* = 35, 38.0%) met less than four criteria and were categorized in the “Low UC” subgroup. There were 19 studies (*k* = 20) that were categorized as “Low UC” that met three out of four criteria, of which, 16 studies (*k* = 17) failed the fourth criterion of having nonresearch therapists deliver the treatment.

Regarding outcomes, a total of 68 effect sizes measuring reduction in SDV were yielded from 61 studies which reported an average of 1.48 SDV outcomes (SD = 0.62, range 1–3). A total of 34 effect sizes measuring reduction in CSU were yielded from 29 studies which reported an average of 2.24 CSU outcomes (SD = 1.24, range 1–6). Lastly, a total of 43 effect sizes measuring reduction in SI were yielded from 39 studies which reported an average of 1.13 SI outcomes (SD = 0.41, range 1–3). Table 2 shows the final sample of studies and effect sizes broken down by UC subgroup, effect type, and outcome.

**Table 2:** Study sample by UC subgroup, effect type, and outcome.

	*n*	*k*	Controlled study effects (*k* = 30)			Uncontrolled study effects (*k* = 62)		
			SDV	CSU	SI	SDV	CSU	SI
Full sample	83	92	23	13	17	45	21	26
UC Subgroup								
Low	31	35	11	3	7	19	9	8
High	52	57	12	10	10	26	12	18

Abbreviations: CSU = crisis service use, *k* = number of effects, *n* = number of studies, SDV = self‐directed violence, SI = suicidal ideation, UC = usual care.

Studies took place primarily in outpatient settings (*k* = 77, 83.7%) with fewer studies set in context of inpatient (*k* = 5, 5.4%), intensive outpatient (*k* = 4, 4.3%), partial hospitalization program (*k* = 1, 1.1%), long‐term inpatient/residential programs (*k* = 2, 2.2%), and a combination of settings (*k* = 3, 3.2%). Across studies, participant sample sizes ranged from 6 to 250 with a mean of 48.8 (SD = 44.7) totaling 4489 individuals. On average, 28% (SD = 18%) of participants discontinued the study treatment prematurely with an average of 19% (SD = 15%) participants attritting from the study data collection prior to study completion. The average participant was female (*M* = 86.6%), aged 27.1 years (SD = 8.65, range 10‐65). See Table 3 for a summary of the participant information reported across studies.

**Table 3:** Summary of study‐reported participant information.

Participant demographics	*k* (%)
Age	
Adolescents only	24 (26.1)
Adults only	57 (62.0)
Combination of Adults and Adolescents	10 (10.9)
NR	1 (1.1)
Race	
Majority white/non‐aboriginal	41 (44.6)
Majority non‐white/aboriginal	5 (5.4)
NR	46 (50.0)
Sample Psychopathology	M^k^ (SD)
Borderline personality disorder^a^	86.1% (28.0%)
Any anxiety disorder^b^	49.4% (25.3%)
Any depressive/mood disorder^c^	65.4% (22.0%)
Post‐traumatic stress disorder^d^	41.7% (27.4%)
Any substance use/abuse disorder^e^	33.4% (20.1%)
Lifetime History of Engagement in Outcomes	
Unspecified self‐directed violence^f^	92.8% (14.8%)
Suicide attempt^g^	70.1% (28.6%)
Non‐suicidal self‐injury^h^	85.3% (18.3%)
Crisis service use^j^	71.3% (19.8%)

Abbreviations: *k* = number of effects, NR = not reported.

a *k* = 64.

b *k* = 38.

c *k* = 51.

d *k* = 35.

e *k* = 45.

f *k* = 45.

g *k* = 27.

h *k* = 31.

j *k* = 25.

k Mean percentage across studies of participants reported within the respective participant characteristic.

The DBT intervention provided across studies varied widely. All four core components of DBT (individual, skills group, phone coaching, and therapist consultation team) were provided in most cases (*k* = 56, 60.9%). Of the four components, skills group was most frequently provided (*k* = 91, 98.9%), followed by individual (*k* = 81, 88.0%), therapist consultation team (*k* = 76, 82.6%), and phone coaching (*k* = 61, 66.3%). Less frequently, the pre*k*treatment goal setting and orientation component of DBT was provided (k = 45, 48.9%). Treatment duration was bimodal with most DBT conditions receiving treatment for approximately 12 months (*k* = 39, 42.4%) or 6 months (*k* = 26, 28.2%). In 24 cases (26.1%), additional DBT‐oriented services were provided as part of the study protocol, which most often included family and parent sessions as needed (in alignment with DBT‐A protocol), additional skills practice groups, and milieu therapy in inpatient and residential settings. In 40 cases (43.5%), additional non‐DBT services were provided as part of the study protocol, which most often took the form of psychiatric medication management, case management, and unspecified individual and group therapies.

As indicated by one of the UC criteria, participating therapists were employed clinical staff at the settings in which the study took place in 61 cases (66.3%). The educational and training backgrounds were reported for 74 cases (80.4%) and included mostly multidisciplinary teams (*k* = 46, 50.0%) and teams of providers with psychology training backgrounds with varying levels of education including (*k* = 17, 18.5%) and excluding unlicensed trainees (*k* = 11, 12.0%). The training received by participating therapists in DBT also varied, with an average of 67.5 h spent in training (SD = 47.9) among the cases that reported this information (*k* = 49; 53.2%).

The most common form of therapist training was the intensive training model (*k* = 30, 32.6%; ITM; Landes and Linehan 2012). In 16 cases (17.4%), unofficial study‐specific methods were employed for training which varied in duration. In 13 cases (14.1%), there was an unspecific reference to training but no details provided as to what training entailed, from whom it was received, or the duration. In eight cases (8.7%), the therapists received varying levels of training, with some unspecified portion receiving intensive training. In 28 cases, (30.4%) therapists received some form of support in the implementation of DBT which included additional weekly generic supervision (*k* = 5, 5.4%), weekly supervision with a provider with expertise in DBT (*k* = 6, 6.5%), weekly supervision with an official DBT trainer (*k* = 4, 4.3%), brief consultation with an official DBT trainer (*k* = 3, 3.2%), ongoing consultation with an official DBT trainer (*k* = 5, 5.4%), ongoing consultation with a provider with expertise in DBT (*k* = 2, 2.2%), and regular feedback guided by DBT adherence measurement (*k* = 3, 3.2%).

Treatment adherence was not mentioned in most cases (*k* = 51, 55.4%) and it was informally monitored or referenced in 16 cases (17.4%). Adherence data were formally tracked and reported in over one quarter of cases (*k* = 25, 27.2%). Of those 25 cases that reported adherence, the majority (*k* = 22) referenced the use of an unpublished measure under Dr. Linehan's name with various dates and co‐authors that was scored on a 5‐point Likert scale in which a score of four or greater is considered adherent. Various names were used to reference these scales including DBT Adherence Coding Scale, DBT Adherence Rating Measure, DBT Adherence Scale, DBT Expert Rating Scale, and DBT Global Rating Scale. Sixteen of the 22 reported mean scores greater than four (range 4.00–4.40). Six reported mean scores slightly below four (range 3.80–3.92) and noted that the mean score for the large majority of cases was above four. Three studies used study‐specific measures to report adherence (Andreasson et al. 2016; Pasieczny and Connor 2011; Wetterborg et al. 2020).

### 3.2 Pooled Effects of Controlled Studies in Usual Care

The model examining DBT's effectiveness in reducing SDV in comparison to non‐DBT comparison conditions in UC included 12 studies and revealed an overall significant pooled difference in effect size between DBT and non‐DBT comparison conditions (*g* = 0.50, CI 0.10 to 0.89, *p* = 0.02). The between‐study heterogeneity was substantial with an *I* ^2^ value of 98.3% (CI: 97.8% to 98.7%) suggesting that a large majority of the variance was attributable to between‐study differences. The CSU model examining controlled studies in UC included 10 studies and revealed a non‐significant pooled difference in effect size between DBT and non‐DBT comparison conditions (*g* = 0.09, CI: −0.25 to 0.43, *p* = 0.57). The between‐study heterogeneity was moderate with an *I* ^2^ value of 68.1% (CI: 38.3% to 83.5%). The SI model examining controlled studies in UC included 10 studies and revealed a nonsignificant pooled difference in effect size between DBT and non‐DBT comparison conditions (*g* = 0.22, CI: −0.25 to 0.68, *p* = 0.31). The between‐study heterogeneity was substantial with an *I* ^2^ value of 83.9% (CI: 72.0% to 90.8%).

### 3.3 Pooled Effects of Uncontrolled Studies in Usual Care

The three‐level hierarchical model examining the magnitude of DBT's effectiveness in the subset of uncontrolled studies conducted in UC revealed a large reduction in SDV in 26 effect sizes among 23 studies (*g* = 0.81, CI: 0.60 to 1.02, *p* < 0.0001). The overall *I* ^2^ value for the model was moderate at 71.0% with 14.9% and 56.1% occurring at levels two and three, respectively. A comparison of the full three‐level model with the reduced two‐level model indicated the two‐level model (AIC = 40.88, BIC = 43.33) had a slightly better fit than the three‐level model (AIC = 42.33, BIC = 45.99) but that the models were not significantly different (*χ* ^2^ = 0.55, *p* = 0.46). This suggests that the findings within studies were largely homogeneous.

The three‐level hierarchical model examining the magnitude of DBT's effectiveness in the subset of uncontrolled studies conducted in UC revealed a large reduction in CSU in 12 effect sizes among 11 studies (*g* = 0.60, CI: 0.35 to 0.85, *p* = 0.0003). The overall *I* ^2^ value for the model was moderate at 73.1%, all of which occurred at level three. A comparison of the full three‐level model with the reduced two‐level model indicated the three‐level model (AIC = 13.88, BIC = 15.08) had a slightly better fit than the reduced two‐level model (AIC = 15.09, BIC = 15.89) but that the models were not significantly different (*χ* ^2^ = 3.21, *p* = 0.07), providing support for the application of the additional parameter in the full model.

The three‐level hierarchical model examining the magnitude of DBT's effectiveness in the subset of uncontrolled studies conducted in UC revealed a large reduction in SI in 18 effect sizes among 16 studies (*g* = 0.68, CI: 0.53 to 0.83, *p* < 0.0001). The overall *I* ^2^ value for the model was moderate at 54.3%, all of which occurred at level two. A comparison of the full three‐level model with the reduced two‐level model indicated the reduced two‐level model (AIC = 11.60, BIC = 13.27) had a slightly better fit than the full three‐level model (AIC = 13.60, BIC = 16.10) but the models were not significantly different (*χ* ^2^ = 0.00, *p* = 1.00). This suggests that the outcomes within studies were largely homogeneous.

### 3.4 Publication Bias

Egger's regressions were conducted for all six models. Funnel plot asymmetry was indicated for two of the six models: the SDV model for controlled studies in UC (*t*(10) = −6.66, *p* < 0.0001) and the CSU model for uncontrolled studies in UC (*t*(10) = 2.95, *p* = 0.0147). After the inclusion of seven hypothetically missing studies, the pooled effect of controlled studies of DBT's reduction of SDV in UC remained significant (*g* = 1.63, CI: 0.85 to 2.41, *p* = 0.0003; see Material S1 for further details). For the uncontrolled CSU model with all studies, an additional five hypothetically missing studies were imputed, resulting in a nonsignificant pooled effect of (*g* = 0.29, CI: −0.02 to 0.60, *p* = 0.0646). Sensitivity analyses revealed that effect sizes did not significantly differ on the basis of being a dissertation, use of randomization, or attrition levels greater than 40% (all *p* > 0.05).

### 3.5 Meta Regressions

Table 4 summarizes the results of six meta‐analytic regression models that were run with the UC subgroups indicated as a moderator. None of the models revealed significant differences between studies categorized as high in UC and studies categorized as low in UC. Additional meta‐regression analyses examined the degree to which effect sizes differed based on theoretical and exploratory predictors (see Table 5). For the controlled studies reporting SDV outcomes, the number of DBT intervention components (*b* = −0.35, SE = 0.15, *p* = 0.034), and therapist training hours (*b* = −0.01, SE = 0.004, *p* = 0.015) both emerged as significant predictors in the individual models. Notably, the effect was in the opposite direction than anticipated for each. However, when entered into the model simultaneously, neither predictor remained significant. None of the predictors emerged as significant in individual or combined models for the controlled studies reporting CSU or SI outcomes. For the uncontrolled studies reporting SDV outcomes, only therapist training emerged as a significant predictor, indicating that the hours spent by therapists in DBT training were positively associated with effect sizes. For the uncontrolled studies reporting CSU outcomes, only the variable indicating whether the study reported data on adherence emerged as a significant predictor, indicating that having reported data on treatment adherence was positively associated with effect sizes. Finally, for uncontrolled studies reporting SI outcomes, only the setting variable emerged as a significant predictor, indicating a positive association between studies set in an outpatient context and effect sizes. Intervention dose and implementation support did not emerge as significant predictors for any of the models.

**Table 4:** Subgroup analyses examining usual care as a moderator.

Outcome	UC subgroup	Controlled studies					Uncontrolled studies				
		*n*	Hedges' *g* (95% CI)	*P_A_*	*Q_B_*	*P_B_*	*k* (*n*)	Hedges' *g* (95% CI)	*P_A_*	*Q_B_*	*P_B_*
SDV					668.06	< 0.001				117.89	< 0.001
	Low UC	11	0.44 (0.08–0.81)	0.019			19 (16)	0.68 (0.42–0.94)	< 0.001		
	High UC	12	0.50 (0.01–0.99)	0.815			26 (22)	0.81 (0.48–1.14)	0.430		
CSU					28.90	0.002				74.10	< 0.001
	Low UC	3	0.13 (−0.57–0.83)	0.691			9 (6)	0.89 (0.51–1.26)	< 0.001		
	High UC	10	0.09 (−0.68–0.84)	0.899			12 (10)	0.61 (0.15–1.07)	0.221		
SI					70.05	< 0.001				54.64	< 0.001
	Low UC	7	0.27 (−0.18–0.73)	0.221			8 (6)	0.96 (0.67–1.25)	< 0.001		
	High UC	10	0.21 (−0.40–0.82)	0.828			18 (16)	0.69 (0.35–1.02)	0.100		

*Note:* The sample size for controlled studies is represented by n (studies) as there was only one effect per study. The sample size for uncontrolled studies is represented by *k* effects that were included in the sample within n clusters (studies) due to the hierarchical structure of the data.

Abbreviations: CI = confidence interval, CSU = crisis service use, *k* = number of effects, *n* = number of studies, *P_A_* = subgroup effect on outcome, *P_B_* = heterogeneity among subgroups, SDV = self‐directed violence, SI = suicidal ideation, UC = usual care.

**Table 5:** Meta‐regression models.

Model	Predictor	Coefficient (95% CI)	*t*	*p* value	τ^2^	*I* ^2^	*R* ^2^
CON‐SDV^a^					0.06	50.18%	51.18%
	(Intercept)	1.77 (0.13–3.41)	2.35	0.037			
	DBT Components	−0.27 (−0.77–0.23)	−1.19	0.258			
	Therapist Training	−0.007 (−0.02–0.00)	−1.61	0.133			
UNC‐SDV^b^					0.10	60.07%	25.17%
	(Intercept)	0.55 (0.26–0.00)	3.89	0.001			
	Therapist Training	0.004 (0.00–0.01)	2.56	0.018			
UNC‐CSU^c^					0.13	75.28%	25.73%
	(Intercept)	0.60 (0.37–0.82)	5.57	< 0.001			
	Adherence Reported	0.73 (0.16–1.30)	2.69	0.015			
UNC‐SI^d^					0.04	46.02%	39.49%
	(Intercept)	0.33 (−0.08–0.75)	1.66	0.109			
	Outpatient Setting	0.46 (0.02–0.90)	2.18	0.039			

Abbreviations: CON = controlled studies, CSU = crisis service use, DBT = Dialectical Behavior Therapy; SDV = self‐directed violence, SI = suicidal ideation, UNC = uncontrolled studies.

^a^ *n* = 15.

^b^ *n* = 23.

^c^ *n* = 21.

^d^ *n* = 26.

## 4 Discussion

The current study found that DBT demonstrated a large effect (*g* = 0.81) in reducing SDV and medium effects in reducing CSU (*g* = 0.60) and SI (*g* = 0.68) in uncontrolled studies. DBT was significantly more effective than comparison conditions in reducing SDV with a medium effect size (*g* = 0.50), whereas effects for CSU and SI in controlled studies were small and nonsignificant. Notably, the effect size for CSU was no longer significant after adjusting for publication bias and effect size adjustment also altered the magnitude of the controlled SDV effect (*g* = 1.63). Taken together, these findings suggest that support for DBT is strongest and most consistent for reductions in SDV and evidence for CSU and SI is more limited and should be interpreted with greater caution. Moderate to substantial between‐study heterogeneity was observed across all models, indicating considerable variability in effect sizes across studies. This heterogeneity likely reflects differences in study design, populations, intervention delivery, and outcome measurement, and limits the extent to which pooled estimates can be interpreted as representing a single, consistent treatment effect. As such, findings should be understood as reflecting average effects across diverse implementations of DBT rather than uniform outcomes. Effect sizes did not differ between studies conducted in UC and non‐UC settings. Among uncontrolled studies, therapist training was associated with greater reductions in SDV, reporting of adherence was associated with greater reductions of CSU, and outpatient settings were associated with greater reductions in SI.

The association between therapist training and reduction in SDV in uncontrolled studies aligns with prior work demonstrating fewer suicide attempts among clients treated by more highly trained DBT providers (Pasieczny and Connor 2011). This finding underscores the potential value of investing in therapist training. Additionally, given the costliness of the intensive training program and preliminary evidence that less intensive training models can be effective (Dimeff et al. 2009, 2011), it is also important to understand how the various DBT training models differentially predict specific therapist behaviors associated with a reduction in SDV. For example, one study found that DBT therapists with intensive training were more likely to review diary cards and discuss the treatment target hierarchy (which traditionally prioritizes self‐harm behaviors) than therapists who received shorter, less intensive trainings (DiGiorgio et al. 2010). These findings suggest that diary card review and/or discussions of target hierarchy may be mechanisms through which therapist training can affect clinical outcomes. Further research is needed to replicate these findings and to identify other potential mechanisms that can be emphasized in less intensive training models.

Although adherence could not be directly evaluated due to inconsistent reporting, studies that reported adherence were associated with greater reductions in CSU. Given that the studies reporting adherence were largely above the stated cutoffs for what was considered adherent, these results are consistent with prior findings that within community therapists, higher adherence was associated with reduced frequency of hospitalizations (Harned et al. 2022). Coding for therapist adherence via direct observation can be time consuming and costly (Becker‐Haimes et al. 2022). Thus, it is possible that lack of resources served as a confound in the relationship between studies' ability to formally code for adherence and effects. Practically, this finding suggests that the exercise of adherence monitoring and reporting may serve as a useful tool in supporting treatment outcomes. For settings in which resources for formal adherence coding are not available, the implementation of a system for adherence monitoring and reporting may be beneficial.

Uncontrolled studies conducted in outpatient settings demonstrated greater reductions in SI than those conducted in higher levels of care. However, higher levels of care were underrepresented in the current sample and generally much shorter in duration than outpatient settings. Although DBT prioritizes the reduction of self‐harm behaviors as a target of treatment, this may be particularly emphasized in higher levels of care given that stabilization tends to be the primary goal of the setting regardless of the type of treatment. Research with adolescents has found that there are distinct trajectories in SI following inpatient hospitalizations, the most common of which involves significant SI at baseline that tends to decline between 3 and 6 months following discharge (Prinstein et al. 2008; Wolff et al. 2018). Thus, it is possible that higher levels of care are comparably effective in reducing SI, but that these results may be better observed at later time points.

The finding that DBT was not significantly more effective in reducing CSU and SI than control conditions further contributes to a mixed literature. Specifically, DeCou et al. (2019) meta‐analysis of controlled DBT evaluations yielded a significant pooled estimate of *d* = −0.38, whereas, Fox et al. (2020) conducted a meta‐analysis of interventions for suicidality‐related outcomes which included subgroup analysis of intervention types and found that DBT yielded a non‐significant weighted mean risk ratio of 0.65. With respect to SI, the current findings are consistent with those of DeCou et al. (2019), who did not find a significant reduction of SI. Yet, Kothgassner et al. (2020, 2021) found that DBT‐A produced moderate reduction of SI in adolescent samples. Importantly, results do not necessarily indicate that DBT was ineffective in reducing these outcomes altogether, but rather that the comparison conditions, many of which involved active treatments, were similarly effective. There was high variability among comparison conditions, which included active alternative treatments designed to intervene on the same sets of behaviors, active treatment as usual conditions in which clients were regularly receiving non‐DBT psychological services facilitated by the study, and passive treatment as usual conditions in which clients were provided referrals or given the option to seek services elsewhere. Future research should examine differential effects between active and passive treatment as usual conditions. Prior work by Gregory and Sachdeva (2016) suggests that ongoing engagement with a mental health provider, regardless of treatment modality, is valuable. It has also been found that a substantial portion of individuals discharged to community‐based care following an ED visit (similar to a passive TAU condition) do not receive follow up care (Bridge et al. 2012). Further, in the case that DBT and alternative interventions for suicidality yield comparable outcomes, future work should consider an economic analysis weighing the relative cost of each in real‐world settings.

Subgroup analyses did not detect any meaningful differences between studies conducted in UC settings and studies not conducted in UC settings. The UC criteria used in previous meta‐analyses (Michelson et al. 2013; Weisz et al. 1995) may not optimally capture meaningful distinctions in the context of DBT. For example, in all cases that criterion two was met, criterion three was also met, suggesting overlap between these dimensions and that they are not independently informative. Moreover, many studies classified as “low UC” differed from “high UC” studies on only a single criterion, most commonly therapist type, further limiting the discriminative utility of the UC variable. Taken together, these findings suggest that the UC construct as operationalized in the current study, may not function as a strong indicator of differences between research and real‐world contexts in DBT. As such, the absence of significant UC effects should not be interpreted as evidence of equivalence across settings, but may instead reflect limitations in the operationalization of UC. Future work may benefit from incorporating additional system‐level factors that better capture variation in real‐world DBT delivery, such as differences between service delivery constraints of CMHCs and specialty DBT programs or hybrid specialty care settings, as well as differences in funding and insurance structures, as suggested by Cummings et al. (2017).

Several limitations merit consideration. First, substantial heterogeneity was observed across studies, likely reflecting variability in study design, populations, intervention delivery, and outcome measurement. Though several sources of heterogeneity were identified a priori and examined as predictors, additional sources of heterogeneity emerged during the review process. For example, there were observed differences in how outcomes were defined and measured and variability in the timelines upon which they were evaluated, the treatment doses received likely differed from that which was prescribed, several studies offered additional DBT‐related services that were challenging to quantify, and participants varied greatly on several characteristics (e.g., age, psychopathology, medications, baseline severity). As a result, pooled estimates should be interpreted as average effects across diverse implementations of DBT rather than as reflecting a single, consistent treatment effect. Second, given the variability in DBT program delivery, particularly in the context of routine care settings where resource constraints may necessitate adaptation, the present findings should be interpreted as reflecting DBT as implemented across diverse clinical contexts rather than a uniform, fully adherent DBT protocol. Third, to create definitional consistency, included interventions were required to teach all four DBT skills modules. Though this decision strengthened confidence that the interventions reflected DBT content rather than more loosely DBT‐informed care, it also likely increased representation of studies in which skills acquisition was a central treatment mechanism. Consistent with this possibility, nearly all interventions included incorporated a skills group component. Thus, the present findings may be particularly reflective of DBT implementations that emphasize formal skills training and findings may not fully generalize to more partial or otherwise modified implementations of DBT.

Fourth, statistical power available for moderation analyses was limited. Although the overall meta‐analysis included 83 studies, the number of studies contributing to each moderation model was substantially smaller due to stratification by outcome and study design (see Table 2). Accordingly, null findings in the moderation analyses should also be interpreted with caution, as they may reflect insufficient power rather than the absence of meaningful effects. Future research with larger and more homogenous samples may be better positioned to detect and clarify these relationships. Fifth, combining the behavioral outcomes of non‐suicidal self‐injury and suicide attempts into one category of SDV limits the ability to draw conclusions about DBT's effectiveness in reducing these behaviors independently. Although these behaviors share common risk factors and are jointly prioritized as treatment targets in DBT, they differ in intent, lethality, and clinical course. Future work may benefit from disaggregating these outcomes to better understand potential differential effects of DBT across forms of self‐directed harm. Finally, the most recent literature search was conducted in September 2022, and studies published thereafter were not included, which may limit the currency of the findings, particularly given the rapidly evolving DBT literature.

## Funding

The authors have nothing to report.

## Conflicts of Interest

The authors declare no conflicts of interest.

## Supporting information


Supporting File

## Data Availability

The data that support the findings of this study are available from the corresponding author upon reasonable request. Data sharing is not applicable to this article as no new data were created or analyzed in this study. Procedures were preregistered with PROSPERO (CRD42021270325).
