# Supplementary material for: The Effectiveness of Dialectical Behavior Therapy in Reducing Self‐Directed Violence and Suicidality in Usual Care Settings: A Meta‐Analysis
Source: J Clin Psychol. 2026 Aug 1;82(11):1699–743. doi: 10.1002/jclp.70195 (PMC13630350; doi:10.1002/jclp.70195)
Supplement: Supplementary file 1 [file JCLP-82-1699-s001.docx]

Supplemental Material S1: Publication Bias in the SDV Model of Controlled Studies in UC

In the model estimating the reduction of SDV among controlled studies in UC, the effect for DBT increases rather substantially when taking into account publication biases. The estimation before adjusting for publication bias is 0.50 (CI 0.10 to 0.89, p=0.02). After the inclusion of seven hypothetically missing studies, the pooled effect increased to 1.63 and remained significant (CI 0.85 to 2.41, p=.0003).

One likely explanation for this discrepancy is that an outlier with a greater effect than the rest of the studies appears to have influenced the trim and fill correction. The “dmetar” package was used to identify outliers and Priebe et al., 2012 was identified. A sensitivity analysis was conducted where the trim and fill procedures were applied with the outlier excluded. This resulted in only one hypothetical study being imputed and a pooled estimate of 0.28 (CI -0.03 to 0.59, p=0.07).

A visual representation comparing the two approaches appears below. A summary of the effects for each approach can be found at the end of this supplement.

One additional note: The outlier article (Priebe et al., 2012) required a unique approach to effect size estimation as the outcome data were provided in the form of IRRs estimated Because the data provided was in the form of incidence rate ratios (IRRs). The following procedure was used:

EST = log (IRR)

SE = (Log(LL of CI) - EST) /(-1.96)

T-statistic = EST / SE

That information was input into this calculator to calculate d: <https://lbecker.uccs.edu/>

*Summaries of the trim and fill adjustments with all data included (left) and with outlier excluded (right)*
